# Reassessment-stage metabolic indices and subsequent cardiorenal outcomes in adults with diabetes: CHARLS and an independent hospital validation cohort

**DOI:** 10.3389/fendo.2026.1926018

**Published:** 2026-08-28

**Authors:** Mingliang Liu, Yan Kong, Shihang Chen, Shi Wu, Bei Sun, Liming Chen

**Affiliations:** 1 NHC Key Laboratory of Hormones and Development, Tianjin Key Laboratory of Metabolic Diseases, Chu Hsien-I Memorial Hospital and Tianjin Institute of Endocrinology, Tianjin Medical University, Tianjin, China; 2 School of Medicine, Nankai University, Tianjin, China

**Keywords:** cardiorenal risk, diabetes mellitus, independent validation cohort, insulin resistance, METS-IR, TCBI, triglyceride-glucose index

## Abstract

**Background:**

Simple metabolic indices may capture residual cardiorenal risk beyond conventional glycemic markers, but their reassessment-stage value and external reproducibility remain unclear. We evaluated the triglyceride-glucose (TyG) index, metabolic score for insulin resistance (METS-IR), triglyceride total cholesterol body weight index (TCBI), and cholesterol-high-density-lipoprotein-glucose (CHG) index in relation to subsequent cardiorenal outcomes.

**Methods:**

The CHARLS cohort comprised participants with diabetes identified in 2011 who attended the 2015 survey, remained free of overt cardiorenal outcomes, and had post-2015 follow-up. The primary outcome was the first cardiovascular or kidney event. Analyses included survey-wave-based Cox and discrete-time models, Kaplan-Meier curves, restricted cubic splines, selection weighting, proportional-hazards diagnostics, 10-fold cross-validated calibration and decision curves, and replication in an independent hospital validation cohort.

**Results:**

The eligible CHARLS reassessment cohort included 1,104 participants and 311 composite cardiorenal outcomes; glycemia-adjusted complete-case Cox analyses included 787–800 participants. Each 1-SD increase in TyG, TCBI, and METS-IR was associated with higher risk, with HRs (95% CIs) of 1.26 (1.06-1.49), 1.19 (1.03-1.37), and 1.20 (1.04-1.38), respectively, whereas CHG was not significant. Selection-weighted and discrete-time estimates were similar, and proportional-hazards tests showed no violations. In the wave-approximated 4-year prediction sample (N = 749; 173 events), apparent AUC gains were small and not statistically significant; 10-fold cross-validated AUC increased only from 0.665 to 0.669-0.671, without a consistent decision-curve advantage. In the independent hospital validation cohort (N = 1,578; 296 events), with post-landmark follow-up administratively censored at 4 years, positive composite-outcome associations were reproduced for TyG, TCBI, and METS-IR, whereas CHG showed a weaker and graphically inconsistent pattern.

**Conclusions:**

Among adults with diabetes identified in 2011 who returned for the 2015 CHARLS reassessment and remained free of overt cardiorenal outcomes, higher TyG, TCBI, and METS-IR were associated with subsequent composite cardiorenal outcomes. Robustness analyses supported these associations, whereas predictive improvement was limited. These indices may serve as exploratory, low-burden adjunctive markers at reassessment, with supportive independent clinical validation; clinical utility requires further study.

## 1 Introduction

Diabetes mellitus, predominantly type 2 diabetes, remains a major and rapidly growing global health challenge. According to the 11th edition of the International Diabetes Federation Diabetes Atlas, the global prevalence of diabetes among adults aged 20–79 years reached 11.1%, corresponding to approximately 589 million people, in 2024 and is projected to increase further over the coming decades (1). China carries one of the largest national burdens of diabetes, with recent nationally representative data showing that the age-standardized prevalence of diabetes among Chinese adults reached 13.7% by 2023 (2). Cardiovascular disease and kidney disease are among the most clinically important complications of diabetes and frequently coexist, interact, and accelerate each other’s progression (3–6). This interconnection is increasingly recognized within integrated cardiorenal and metabolic frameworks, which emphasize the shared metabolic and vascular mechanisms underlying diabetes-related cardiovascular and renal complications (5, 6). Accordingly, contemporary guidelines from the American Diabetes Association and Kidney Disease: Improving Global Outcomes emphasize repeated evaluation and integrated management of cardiovascular and kidney risk in people with diabetes (7–10).

Despite advances in glucose-lowering therapy and multifactorial risk control, conventional glycemic markers such as fasting blood glucose (FBG) and HbA1c do not fully capture insulin resistance, atherogenic dyslipidemia, adiposity-related metabolic burden, or residual cardiovascular-kidney-metabolic risk (11, 12). Direct measurement of insulin resistance is costly and impractical for routine clinical practice and large-scale epidemiologic studies, which has prompted interest in non-insulin-based metabolic indices calculated from routinely available clinical measurements (13–20). The triglyceride-glucose (TyG) index, derived from fasting triglycerides and glucose, has been proposed and validated as a practical surrogate of insulin resistance (13–15). The metabolic score for insulin resistance (METS-IR) incorporates fasting glucose, triglycerides, HDL-C, and body mass index to reflect broader cardiometabolic dysregulation (16). The triglyceride total cholesterol body weight index (TCBI) has been evaluated as a simple nutritional-metabolic prognostic marker in cardiovascular settings (17, 18), whereas the cholesterol-HDL-glucose (CHG) index has recently been introduced as a lipid-glucose composite marker for diabetes identification and cardiovascular risk prediction (19, 20).

Accumulating evidence has linked these metabolic indices to cardiovascular and renal outcomes. Higher TyG levels have been associated with incident cardiovascular disease, mortality, chronic kidney disease, and diabetic nephropathy in general populations and diabetes cohorts (21–26). METS-IR has been associated with adverse cardiovascular outcomes in patients with type 2 diabetes and ischemic cardiomyopathy and has also been studied in relation to incident chronic kidney disease (27, 28). TCBI has shown prognostic value in coronary artery disease and heart failure cohorts (17, 18), and CHG has been reported to predict cardiovascular disease risk in population-based data (20). However, previous studies have often focused on single cardiovascular or renal endpoints, baseline measurements, general populations, or selected cardiovascular cohorts. Less is known about whether these indices can stratify subsequent composite cardiorenal risk among adults with previously identified diabetes who remain free of overt cardiorenal outcomes at a later reassessment visit.

Cardiorenal risk in diabetes is dynamic. During the course of diabetes care, body weight, lipid profiles, blood pressure, kidney function, medication use, and lifestyle factors may change substantially, and a single historical measurement may not adequately represent a patient’s current risk state. Periodic reassessment is central to guideline-directed diabetes and kidney care (7–10), and repeated risk assessment has been shown to provide additional prognostic information beyond a single baseline evaluation (29). Conceptually, a reassessment-stage framework reflects the clinical process of updating risk among individuals who remain event-free and at risk at a meaningful follow-up time point. Therefore, evaluating metabolic indices at reassessment may better reflect contemporary insulin-resistance burden and cardiovascular-kidney-metabolic status, thereby informing subsequent risk of cardiorenal outcomes.

Accordingly, using the China Health and Retirement Longitudinal Study (CHARLS), we examined adults with diabetes who were free of overt cardiorenal outcomes at the 2015 reassessment and evaluated the associations of reassessment-stage TyG, CHG, TCBI, and METS-IR with subsequent composite cardiorenal outcomes (30, 31). We further compared these indices with conventional glycemic markers, assessed their incremental value beyond FBG and HbA1c, evaluated dose-response relationships, discrimination, reclassification, subgroup patterns, and individual cardiovascular and kidney outcomes, and examined whether the main findings could be reproduced in an independent, ethics-approved hospital validation cohort of patients with type 2 diabetes. This study aimed to clarify the potential value of simple metabolic indices as adjunctive markers for reassessment-stage cardiorenal risk stratification in diabetes care, supported by independent clinical validation.

## 2 Methods

### 2.1 Data sources and study design

This study used two data sources. The principal analysis was based on the China Health and Retirement Longitudinal Study (CHARLS), a nationally representative longitudinal survey of community-dwelling adults aged 45 years or older in mainland China. The baseline survey was conducted in 2011-2012, followed by subsequent waves in 2013, 2015, 2018, and 2020. CHARLS includes information on demographic characteristics, socioeconomic status, lifestyle factors, chronic diseases, and blood-based biomarkers (30). Blood collection and biomarker assays were performed in both the 2011 baseline wave and the 2015 wave (31). This design combined a publicly available longitudinal cohort with an independent, non-public, ethics-approved hospital clinical validation cohort.

The present study was designed as a reassessment-stage cohort analysis among adults with diabetes who remained free of overt cardiorenal outcomes at the 2015 reassessment. Metabolic indices measured at the reassessment were used to evaluate subsequent cardiorenal risk. To examine the reproducibility of the main findings, we further used an independent retrospective hospital validation cohort derived from an institutional type 2 diabetes specialty database. The hospital validation cohort included patients with baseline assessments between June 2020 and September 2023 and used de-identified routinely collected clinical data. Hospital follow-up records used for outcome ascertainment were available through June 20, 2025.

### 2.2 Study population

For the principal CHARLS cohort, diabetes in 2011 was defined by any of the following criteria: self-reported diabetes, fasting blood glucose (FBG) ≥126 mg/dL, or HbA1c ≥6.5%. Participants were eligible for the principal analysis if they met the diabetes definition in 2011, attended the 2015 survey, were free of the composite cardiorenal outcome at the 2015 reassessment, and had valid post-2015 follow-up information. A total of 2,343 participants met the diabetes definition in 2011, of whom 1,913 attended the 2015 survey. After excluding participants with prevalent composite cardiorenal outcomes or undetermined cardiorenal status at the 2015 reassessment, 1,206 participants were eligible at reassessment. A further 102 participants without valid post-2015 follow-up data were excluded, leaving 1,104 participants in the principal CHARLS analytic cohort. CHARLS did not provide a uniform etiologic classification of diabetes; given the age range and epidemiologic setting, the cohort predominantly represents type 2 diabetes, but specific secondary causes, including pancreatic disease and polycystic ovary syndrome, could not be systematically identified or excluded. The hospital database comprised clinically diagnosed type 2 diabetes, although detailed etiologic classification was not uniformly available.

Two supplementary CHARLS analyses were conducted to clarify how the analytic framework affected the results. First, among participants with diabetes at the 2011 baseline who were free of composite cardiorenal outcomes at that time, 2011 metabolic indices were used to evaluate incident composite cardiorenal outcomes from 2011 to 2015. Second, among all participants with diabetes at the 2015 survey who were free of composite cardiorenal outcomes at 2015, 2015 metabolic indices were used to evaluate post-2015 outcomes. The sample sizes of these datasets are summarized in **Supplementary Table 1**.

For the independent hospital validation cohort, patients were identified from the institutional type 2 diabetes specialty database. The index date was defined as the baseline assessment date used for metabolic-index calculation, based on the available core metabolic laboratory measurements. Patients with prevalent cardiovascular or kidney outcomes at or before the index date were excluded. To reduce reverse causation and same-encounter outcome misclassification, a 90-day landmark was applied; patients who developed a composite cardiorenal outcome within 90 days after the index date or had follow-up shorter than 90 days were excluded. Among 10,419 patients in the hospital database, 2,819 remained eligible after baseline outcome screening and the 90-day landmark requirement. Patients with missing core metabolic variables, physiologically implausible values, or extreme outliers were then excluded. Final validation analyses were restricted to patients with complete baseline metabolic indices and ascertainable post-landmark outcome information, resulting in 1,578 patients in the hospital validation cohort.

### 2.3 Metabolic indices

Four metabolic indices were evaluated: the triglyceride-glucose (TyG) index, cholesterol-HDL-glucose (CHG) index, triglyceride total cholesterol body weight index (TCBI), and metabolic score for insulin resistance (METS-IR). In CHARLS, glucose and lipid variables were expressed in mg/dL. In the hospital cohort, glucose and lipid measurements originally recorded in mmol/L were converted to mg/dL for index calculation. Body weight was expressed in kg and body mass index (BMI) in kg/m^2^.

The indices were calculated as follows: 

$\text{TyG}=\text{ln}\left[\text{TG }x\text{ FBG}/2\right]$ (13, 14)

$\text{CHG}=\text{ln}\left[\text{TC }x\text{ FBG}/\left(2\;x\text{ HDL}-\text{C}\right)\right]$ (19)

TCBI=TG x TC x body weight/1000 (17)

$\text{METS}-\text{IR}=\text{ln }\left[\left(2\;x\text{ FBG}\right)+\text{TG}\right]x\text{BMI}/\text{ln}\left(\text{HDL}-\text{C}\right)$ (16)

TCBI was natural log-transformed before standardization and modeling. For continuous analyses, all indices were standardized to Z scores and analyzed per 1-standard deviation increase. For quartile analyses, each index was categorized into quartiles according to its distribution in the corresponding analytic sample, with the lowest quartile used as the reference group.

### 2.4 Outcome definitions and follow-up

In the CHARLS cohort, the primary outcome was a composite cardiorenal outcome, defined as the first occurrence of either a cardiovascular event or a kidney event after the 2015 reassessment. Cardiovascular events were defined as incident heart disease and/or stroke, and kidney events were defined as incident kidney disease, based on CHARLS disease-history items (30). Exact onset dates were not available in CHARLS; therefore, event time was approximated by the first survey wave in which any component event was reported. Participants without events were censored at the last survey wave with valid outcome information. Thus, CHARLS time-to-event analyses should be interpreted as survey-wave-based approximations rather than analyses based on exact event dates. Cardiovascular and kidney outcomes were also analyzed separately as component outcomes.

In the hospital validation cohort, cardiovascular and kidney outcomes were identified from diagnosis and follow-up records using a narrow clinical outcome definition. Cardiovascular outcomes included clinically recorded coronary heart disease, angina, myocardial infarction, heart failure, stroke, cerebral infarction, cerebral hemorrhage, ischemic cerebrovascular disease, or transient ischemic attack. Kidney outcomes included diabetic kidney disease stage III-V, chronic kidney disease stage 3-5, chronic renal failure, renal failure, uremia, or dialysis. Isolated proteinuria, albuminuria, nonspecific renal-function abnormality, and historical procedure/status terms without an active outcome diagnosis were not counted as endpoint events. These endpoints reflected clinically recorded diagnoses and were not based on renal histopathology; renal fibrosis and glomerulosclerosis were not directly assessed. The hospital composite outcome was defined as the first occurrence of either a cardiovascular or kidney outcome after the 90-day landmark. Follow-up time was calculated from the landmark to the first event or censoring. Patients without events were censored at the earliest of the last available hospital record or 4 years after the landmark; events after the administrative censoring point were not counted. Hospital follow-up records were available through June 20, 2025.

### 2.5 Covariates

In CHARLS, covariates were selected according to clinical relevance and data availability, including age, sex, smoking status, drinking status, hypertension, marital status, residence, and educational level (30). Primary smoking status was defined as ever having smoked versus never smoking. A sensitivity analysis used current smoking versus not currently smoking; cigarette dose and pack-years were unavailable. BMI was additionally included in selected models. FBG and HbA1c were included when assessing associations beyond conventional glycemic markers.

In the hospital validation cohort, available covariates included age, sex, binary current/ever smoking status, drinking status, hypertension, BMI, FBG, and HbA1c. Smoking dose and pack-years were unavailable. Socioeconomic variables such as marital status, education, and residence were not available and were therefore not included in hospital validation models.

### 2.6 Statistical analysis

#### 2.6.1 Baseline characteristics

Continuous variables are presented as mean +/- standard deviation or median (interquartile range), as appropriate, and categorical variables as n (%). Baseline characteristics were compared between participants with and without subsequent composite cardiorenal outcomes. Continuous variables were compared using Student’s t test or the Wilcoxon rank-sum test, and categorical variables using the chi-square test. For descriptive baseline tables, percentages for variables with missing values were calculated using the number of participants with non-missing data for that variable as the denominator.

#### 2.6.2 Main Cox analyses in CHARLS

Cox proportional hazards models were used as approximate time-to-event models based on survey-wave event timing to evaluate the associations of metabolic indices with subsequent composite cardiorenal outcomes. Continuous analyses were conducted per 1-standard deviation increase, and quartile analyses used the lowest quartile as the reference group. Sequential models were fitted as follows:

Model 1 adjusted for age and sex.

Model 2 additionally adjusted for smoking, drinking, hypertension, marital status, residence, and education.

Model 3 was further adjusted for BMI.

Model 4 was used as the glycemia-adjusted model and adjusted for the covariates in Model 2 plus FBG and HbA1c.

The main interpretation focused on Model 4 as the glycemia-adjusted model, because the study aimed to examine whether metabolic indices provided information beyond conventional glycemic markers. Model 3 and Model 4 were presented separately as BMI-adjusted and glycemia-adjusted models rather than combined into a single maximal adjustment model, because BMI or body weight is a component of, or closely related to, several metabolic indices. The same modeling framework was used for continuous and quartile analyses. Cox analyses used complete cases for the variables required in each model; therefore, model-specific sample sizes and event counts differed from the principal eligible cohort.

#### 2.6.3 Comparison with conventional glycemic markers and incremental association analyses

To compare metabolic indices with conventional glycemic markers, head-to-head Cox analyses were performed for FBG, HbA1c, TyG, TCBI, and METS-IR using the same clinically adjusted model including age, sex, smoking, drinking, hypertension, marital status, residence, and education. Incremental association analyses were then performed by adding TyG, TCBI, or METS-IR individually to a model containing clinical covariates, FBG, and HbA1c.

#### 2.6.4 Kaplan-Meier and restricted cubic spline analyses

Kaplan-Meier curves were plotted according to quartiles of each metabolic index, and differences across quartile groups were evaluated using log-rank tests. Restricted cubic spline analyses were used to examine dose-response relationships and potential nonlinearity. Spline-based Cox models with 3 degrees of freedom were fitted for TyG, TCBI, METS-IR, and CHG. Kaplan-Meier curves and spline-based Cox models used the same survey-wave-based event-time approximation as the main Cox analyses. Both the overall association test and the nonlinearity test were reported (32).

#### 2.6.5 Discrimination and reclassification analyses

Prediction performance was evaluated for a wave-approximated 4-year composite cardiorenal outcome in CHARLS. Because exact event dates were unavailable, the 4-year horizon was approximated using interview years from the post-reassessment survey waves. Participants whose first reported composite outcome occurred within 4 years after the 2015 reassessment were classified as cases, whereas participants observed beyond 4 years without an event, or whose first reported event occurred after 4 years, were classified as non-cases. Participants without sufficient outcome information were excluded. Logistic regression models were constructed for the clinical model and models additionally including FBG, HbA1c, TyG, TCBI, or METS-IR. Apparent discrimination was evaluated using ROC curves and AUC. Changes in AUC, continuous NRI, and IDI were estimated after adding each metabolic index to clinical variables, FBG, and HbA1c (33). To evaluate optimism and calibration, stratified 10-fold cross-validation was used to obtain out-of-fold AUC, Brier score, calibration-in-the-large, calibration intercept, and calibration slope. Calibration plots used deciles of out-of-fold predicted risk, and decision-curve analysis used the same out-of-fold predictions across thresholds of 0.05-0.45 (34). Characteristics of participants included in and excluded from the prediction sample were compared. Bootstrap confidence intervals and empirical P values for NRI and IDI used 500 resamples. All model comparisons used a common complete-case prediction sample.

#### 2.6.6 Subgroup and component outcome analyses

Prespecified subgroup analyses were performed according to age (<65 vs. >=65 years), sex, residence, hypertension, smoking status, drinking status, and HbA1c category (<7.0% vs. >=7.0%). Within each subgroup, associations of TyG, TCBI, and METS-IR with the composite outcome were estimated, and P values for interaction were obtained by adding interaction terms to Cox models based on survey-wave event timing. Separate Cox models were also fitted for cardiovascular and kidney outcomes to evaluate the individual components of the composite outcome.

#### 2.6.7 Hospital validation analyses

In the hospital validation cohort, Cox models evaluated TyG, CHG, TCBI, and METS-IR in relation to composite cardiorenal outcomes during post-landmark follow-up, administratively censored at 4 years. Continuous analyses were conducted per 1-SD increase, and quartile analyses used Q1 as the reference. Model 1 adjusted for age and sex; Model 2 additionally adjusted for smoking, drinking, and hypertension; Model 3 was the BMI-adjusted model; and Model 4 was the glycemia-adjusted model and included Model 2 covariates plus FBG and HbA1c. BMI- and glycemia-adjusted models were presented separately because BMI or body weight was a component of, or closely related to, several indices. Kaplan-Meier, restricted cubic spline, and component-outcome analyses were also performed. Missing smoking, drinking, and hypertension covariates were multiply imputed, and Cox estimates were pooled across 20 imputed datasets using Rubin’s rules. Using exact hospital event and censoring dates, IPCW time-dependent AUCs were estimated at 1, 2, 3, and approximately 4 years after the landmark for the clinical model with FBG and HbA1c and after adding TyG, TCBI, or METS-IR (35).

#### 2.6.8 Robustness and additional analyses

To evaluate selection into the principal reassessment cohort, 2011 baseline characteristics were compared between included and non-included participants using standardized mean differences. A logistic model estimated the probability of entering the final cohort from 2011 age, sex, BMI, hypertension, smoking, drinking, marital status, residence, education, FBG, HbA1c, triglycerides, total cholesterol, and HDL-C. Stabilized inverse-probability-of-selection weights were trimmed at the 1st and 99th percentiles. Weighted Cox models used robust standard errors, and balance and effective sample size were reported (36).

Because CHARLS event occurrence was assessed at discrete survey waves, a person-period dataset was constructed for post-2015 intervals and complementary log-log models with interval indicators were fitted as a discrete-time proportional-hazards sensitivity analysis (37). Both unweighted and selection-weighted models used the glycemia-adjusted covariate set. The Cox proportional-hazards assumption was evaluated using Schoenfeld residuals for each marker and globally.

To examine heterogeneity by diabetes history, participants with diabetes and no overt cardiorenal outcome in 2015 were stratified as having diabetes already identified in 2011 or being newly identified in 2015, and marker-by-history interactions were tested. Additional exploratory analyses evaluated VAI and LAP in CHARLS, current smoking in place of ever smoking, and marker-by-sex interactions in both cohorts. For men, VAI=[waist circumference/(39.68 + 1.88×BMI)]×(triglycerides/1.03)×(1.31/HDL-C), and for women, VAI=[waist circumference/(36.58 + 1.89×BMI)]×(triglycerides/0.81)×(1.52/HDL-C) (38). LAP was calculated as (waist circumference-65)×triglycerides in men and (waist circumference-58)×triglycerides in women (39), with waist circumference in cm and lipids in mmol/L. VAI and LAP could not be calculated in the hospital cohort because waist circumference was unavailable in the fixed analytic file.

#### 2.6.9 Missing data and analytic sample sizes

In CHARLS, the principal cohort consisted of 1,104 participants with valid post-2015 follow-up information. Baseline descriptive analyses used all available observed data, and percentages were calculated among participants with non-missing values for each variable. Cox models, head-to-head comparisons, spline analyses, component outcome analyses, and prediction analyses were conducted using complete cases for the variables required in each specific analysis. Therefore, sample size and event counts differed across tables and analytic modules. In particular, analyses involving TCBI or METS-IR required body weight or BMI in addition to glucose and lipid variables, and prediction/reclassification analyses required valid wave-approximated 4-year outcome classification and complete predictor information.

In the hospital validation cohort, core metabolic variables and outcome information were not imputed. Smoking, drinking, and hypertension were multiply imputed because they were non-core covariates with 2.8%, 5.4%, and 11.9% missingness, respectively. Twenty stochastic logistic imputed datasets were generated using age, sex, FBG, HbA1c, BMI, and the other available binary covariates as predictors, and estimates were pooled using Rubin’s rules. The different missing-data strategies reflected the data structures: CHARLS had approximately 25%-27% missingness in biomarker-derived indices and used transparent model-specific complete-case analyses, whereas the fixed hospital cohort had complete core metabolic indices and imputation was limited to three non-core covariates. All metabolic indices were standardized separately within each cohort. Cross-cohort comparisons of per-1-SD estimates were therefore interpreted in terms of direction and statistical consistency rather than direct numerical equivalence of effect sizes.

All statistical tests were two-sided, and P<0.05 was considered statistically significant. Data cleaning and cohort construction were performed using Python. Statistical analyses and figure generation were conducted using Python and R.

## 3 Results

### 3.1 Study population selection and baseline characteristics

**Figure 1:**
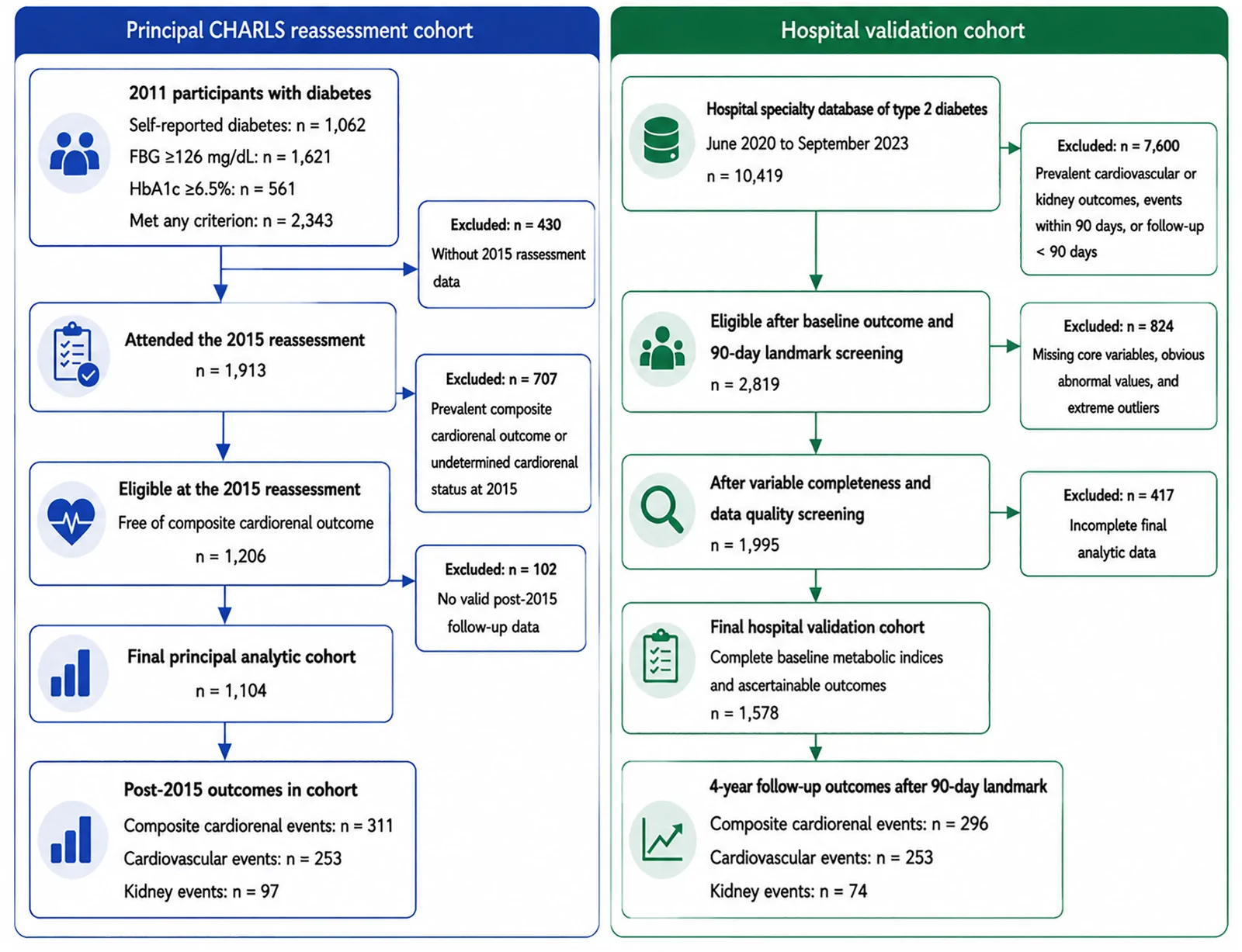
Study flow diagram of the principal CHARLS reassessment cohort and the hospital validation cohort. In the hospital cohort, follow-up began at the 90-day landmark and was censored at the first event, the last available hospital record, or 4 years after the landmark, whichever occurred first. Hospital records were available through June 20, 2025. CHARLS, China Health and Retirement Longitudinal Study; FBG, fasting blood glucose; HbA1c, glycated hemoglobin.

As shown in **Figure 1**, 2,343 participants met the definition of diabetes at the 2011 CHARLS baseline survey based on self-reported diabetes, fasting blood glucose (FBG) ≥ 126 mg/dL, or HbA1c ≥ 6.5%. Among them, 1,913 attended the 2015 reassessment, and 430 were excluded because of missing 2015 reassessment data. A further 707 participants were excluded because of prevalent composite cardiorenal outcomes or undetermined cardiorenal status at the 2015 reassessment. Among the remaining 1,206 participants who were free of composite cardiorenal outcomes at reassessment, 102 were excluded because of no valid post-2015 follow-up data. The final principal CHARLS analytic cohort included 1,104 participants, with 311 composite cardiorenal events, 253 cardiovascular events, and 97 kidney events during post-2015 follow-up. The composition of the supplementary CHARLS analysis datasets is summarized in **Supplementary Table 1**. This cohort represented the principal eligible reassessment cohort for descriptive and follow-up summaries; model-based analyses used complete-case samples defined by the variables required for each specific model.

At the 2011 baseline, participants who entered the final reassessment cohort were younger than those not included (mean 58.56 vs. 62.52 years; standardized mean difference [SMD], -0.419) and less frequently had hypertension (32.0% vs. 53.0%; SMD, -0.424), whereas most metabolic-marker differences were small (**Supplementary Table 9**). Stabilized inverse-probability-of-selection weights trimmed at the 1st and 99th percentiles ranged from 0.576 to 3.144 and yielded an effective sample size of 931. Weighting reduced the absolute SMD for age from 0.215 to 0.034 and for hypertension from 0.216 to 0.016 (**Supplementary Table 10**).

Baseline characteristics of the principal CHARLS cohort are shown in **Table 1**. The mean age was 62.55 ± 8.98 years, 53.0% were women, and 43.2% had hypertension. Participants who subsequently developed composite cardiorenal outcomes were older, more frequently female, and more likely to have hypertension than those without subsequent events. They also had higher BMI, FBG, HbA1c, triglycerides, TyG, CHG, TCBI, and METS-IR values, whereas body weight, total cholesterol, HDL-C, smoking status, drinking status, education, and residence did not differ significantly between groups.

**Table 1:** Baseline characteristics of the principal CHARLS reassessment cohort according to subsequent composite cardiorenal outcome.

Characteristic	Overall	No subsequent cardiorenal outcome	Subsequent cardiorenal outcome	P value
Overall	1104	793	311	
Demographic characteristics				
Age (years)	62.55 ± 8.98	61.94 ± 9.19	64.12 ± 8.24	<0.001
Sex				0.003
Male	519 (47.0%)	395 (49.8%)	124 (39.9%)	
Female	585 (53.0%)	398 (50.2%)	187 (60.1%)	
Education				0.698
Illiterate	519 (47.0%)	368 (46.4%)	151 (48.6%)	
Primary school and below	474 (42.9%)	342 (43.1%)	132 (42.4%)	
Middle school and above	111 (10.1%)	83 (10.5%)	28 (9.0%)	
Residence				0.130
Rural	692 (62.7%)	508 (64.1%)	184 (59.2%)	
Urban	412 (37.3%)	285 (35.9%)	127 (40.8%)	
Lifestyle and clinical characteristics				
Smoking status				0.284
No	625 (56.6%)	441 (55.6%)	184 (59.2%)	
Yes	479 (43.4%)	352 (44.4%)	127 (40.8%)	
Drinking status				0.122
No	591 (53.5%)	413 (52.1%)	178 (57.2%)	
Yes	513 (46.5%)	380 (47.9%)	133 (42.8%)	
Hypertension	465 (43.2%)	287 (37.1%)	178 (58.7%)	<0.001
BMI (kg/m²)	24.23 ± 3.53	23.99 ± 3.44	24.81 ± 3.67	0.002
Body weight (kg)	60.56 ± 11.19	60.32 ± 10.94	61.17 ± 11.82	0.313
Laboratory measurements				
FBG (mg/dL)	113.51 (95.50, 145.95)	111.71 (95.50, 142.34)	117.12 (99.10, 154.95)	0.016
HbA1c (%)	6.40 (5.80, 7.80)	6.20 (5.70, 7.40)	6.70 (6.00, 8.20)	<0.001
Triglycerides (mg/dL)	138.94 (95.58, 211.06)	134.51 (90.71, 194.25)	162.83 (112.39, 238.05)	<0.001
Total cholesterol (mg/dL)	191.30 ± 45.16	190.39 ± 46.68	193.43 ± 41.36	0.355
HDL-C (mg/dL)	49.77 ± 12.07	49.84 ± 11.96	49.60 ± 12.35	0.796
Metabolic indices				
TyG	9.13 ± 0.76	9.07 ± 0.75	9.28 ± 0.75	<0.001
CHG	5.49 ± 0.50	5.47 ± 0.50	5.55 ± 0.49	0.033
TCBI	1598.97 (951.83, 2618.14)	1507.30 (885.26, 2464.00)	1839.76 (1130.72, 3125.58)	<0.001
METS-IR	38.04 ± 7.32	37.45 ± 7.13	39.43 ± 7.57	<0.001

Values are presented as mean ± SD, median (IQR), or n (%). P values compare participants with and without a subsequent composite cardiorenal outcome. Percentages were calculated using participants with non-missing data for each variable as the denominator. SD, standard deviation; IQR, interquartile range; BMI, body mass index; FBG, fasting blood glucose; HbA1c, glycated hemoglobin; HDL-C, high-density lipoprotein cholesterol; TyG, triglyceride-glucose index; CHG, cholesterol-high-density lipoprotein-glucose index; TCBI, triglyceride-total cholesterol-body weight index; METS-IR, metabolic score for insulin resistance.

The independent hospital validation cohort was derived from the institutional type 2 diabetes specialty database. After baseline outcome screening, 90-day landmark screening, variable completeness assessment, data quality screening, and ascertainment of post-landmark outcome information, 1,578 patients were included in the hospital validation cohort. During post-landmark follow-up censored at the first event, last available hospital record, or 4 years after the landmark, 296 composite cardiorenal events were observed (**Figure 1**). Baseline characteristics of the hospital validation cohort are presented in **Supplementary Table 7**. Compared with patients without composite cardiorenal outcomes, those with composite cardiorenal outcomes were older and had higher BMI, higher prevalence of hypertension, higher triglycerides, higher UACR, lower eGFR, and higher TyG, TCBI, and METS-IR values. FBG, HbA1c, total cholesterol, HDL-C, smoking status, drinking status, and CHG did not differ significantly between groups.

### 3.2 Associations of metabolic indices with subsequent composite cardiorenal outcomes in the CHARLS reassessment cohort

The associations between metabolic indices and subsequent composite cardiorenal outcomes in the principal CHARLS reassessment cohort are shown in **Table 2**. Because complete covariate information differed across indices and adjustment models, Cox analyses were conducted in model-specific complete-case samples. In the glycemia-adjusted Model 4 analyses, the complete-case samples included 800 participants with 240 events for TyG and CHG and 787 participants with 237 events for TCBI and METS-IR. In these analyses, higher TyG, TCBI, and METS-IR were each associated with a higher risk of composite cardiorenal outcomes. After adjustment for demographic factors, lifestyle factors, hypertension, marital status, residence, education, FBG, and HbA1c, the adjusted HRs per 1-SD increase were 1.26 (95% CI, 1.06-1.49; P = 0.008) for TyG, 1.19 (95% CI, 1.03-1.37; P = 0.015) for TCBI, and 1.20 (95% CI, 1.04-1.38; P = 0.013) for METS-IR. In contrast, CHG was not significantly associated with the composite outcome in the glycemia-adjusted model (HR, 1.00; 95% CI, 0.79-1.27; P = 0.981).

**Table 2:** Associations of metabolic indices with subsequent composite cardiorenal outcomes in the principal CHARLS reassessment cohort.

Categories	Model 1		Model 2		Model 3		Model 4	
	HR (95% CI)	P	HR (95% CI)	P	HR (95% CI)	P	HR (95% CI)	P
TyG								
Continuous	1.27 (1.12, 1.43)	<0.001	1.22 (1.07, 1.40)	0.003	1.21 (1.06, 1.39)	0.006	1.26 (1.06, 1.49)	0.008
Q1	Ref		Ref		Ref		Ref	
Q2	1.60 (1.06, 2.39)	0.024	1.43 (0.95, 2.16)	0.087	1.36 (0.90, 2.07)	0.147	1.40 (0.93, 2.13)	0.110
Q3	1.82 (1.22, 2.72)	0.003	1.56 (1.04, 2.36)	0.033	1.51 (0.99, 2.30)	0.053	1.52 (0.99, 2.32)	0.053
Q4	2.33 (1.58, 3.44)	<0.001	2.02 (1.34, 3.02)	<0.001	1.96 (1.30, 2.95)	0.001	2.04 (1.29, 3.23)	0.002
CHG								
Continuous	1.14 (1.01, 1.28)	0.038	1.10 (0.97, 1.26)	0.133	1.11 (0.97, 1.27)	0.138	1.00 (0.79, 1.27)	0.981
Q1	Ref		Ref		Ref		Ref	
Q2	1.56 (1.06, 2.28)	0.023	1.49 (1.01, 2.19)	0.045	1.40 (0.94, 2.08)	0.101	1.41 (0.95, 2.08)	0.085
Q3	1.50 (1.02, 2.20)	0.040	1.33 (0.89, 1.97)	0.162	1.27 (0.85, 1.91)	0.243	1.17 (0.77, 1.77)	0.456
Q4	1.68 (1.15, 2.47)	0.008	1.50 (1.01, 2.22)	0.045	1.48 (0.99, 2.21)	0.059	1.06 (0.60, 1.88)	0.834
TCBI								
Continuous	1.26 (1.11, 1.43)	<0.001	1.21 (1.05, 1.38)	0.008	1.17 (1.01, 1.36)	0.037	1.19 (1.03, 1.37)	0.015
Q1	Ref		Ref		Ref		Ref	
Q2	1.55 (1.04, 2.32)	0.031	1.38 (0.91, 2.08)	0.131	1.32 (0.87, 2.02)	0.195	1.32 (0.87, 2.00)	0.190
Q3	1.83 (1.23, 2.72)	0.003	1.53 (1.02, 2.29)	0.041	1.43 (0.93, 2.20)	0.103	1.45 (0.96, 2.18)	0.076
Q4	2.18 (1.47, 3.21)	<0.001	1.89 (1.26, 2.83)	0.002	1.76 (1.14, 2.72)	0.011	1.80 (1.20, 2.72)	0.005
METS-IR								
Continuous	1.29 (1.14, 1.46)	<0.001	1.22 (1.07, 1.40)	0.004	1.34 (1.03, 1.74)	0.027	1.20 (1.04, 1.38)	0.013
Q1	Ref		Ref		Ref		Ref	
Q2	0.98 (0.66, 1.46)	0.916	0.88 (0.58, 1.33)	0.549	0.92 (0.59, 1.44)	0.729	0.82 (0.54, 1.25)	0.355
Q3	1.49 (1.03, 2.16)	0.036	1.30 (0.88, 1.92)	0.182	1.42 (0.87, 2.31)	0.157	1.23 (0.83, 1.82)	0.306
Q4	1.95 (1.36, 2.81)	<0.001	1.66 (1.13, 2.44)	0.010	1.89 (1.05, 3.41)	0.033	1.54 (1.04, 2.29)	0.033

Model 1 adjusted for age and sex. Model 2 additionally adjusted for ever smoking, ever drinking, hypertension, marital status, residence, and education. Model 3 included Model 2 covariates plus BMI. Model 4 included Model 2 covariates plus FBG and HbA1c and is referred to as the glycemia-adjusted model. Models 3 and 4 are presented separately because BMI or body weight is a component of, or closely related to, several metabolic indices. Continuous estimates are shown per 1-SD increase; quartile analyses use Q1 as the reference. TCBI was log-transformed before standardization and modeling. Analyses used model-specific complete-case samples. In Model 4, the analytic samples were N = 800 with 240 events for TyG and CHG and N = 787 with 237 events for TCBI and METS-IR. BMI, body mass index; FBG, fasting blood glucose; HbA1c, glycated hemoglobin; SD, standard deviation; Q, quartile; HR, hazard ratio; CI, confidence interval. TyG, triglyceride-glucose index; CHG, cholesterol-high-density lipoprotein-glucose index; METS-IR, metabolic score for insulin resistance.

Quartile-based analyses showed generally similar patterns. Compared with Q1, the highest quartiles of TyG, TCBI, and METS-IR were associated with higher risks of composite cardiorenal outcomes in the glycemia-adjusted Model 4, with HRs of 2.04 (95% CI, 1.29-3.23; P = 0.002), 1.80 (95% CI, 1.20-2.72; P = 0.005), and 1.54 (95% CI, 1.04-2.29; P = 0.033), respectively. The highest quartile of CHG was not significantly associated with the composite outcome in Model 4 (HR, 1.06; 95% CI, 0.60-1.88; P = 0.834).

Selection-weighted Cox estimates remained similar to the unweighted results: HRs per 1-SD increase were 1.26 for TyG, 1.18 for TCBI, and 1.23 for METS-IR, whereas CHG remained null (**Supplementary Table 10**). Discrete-time complementary log-log models, which accounted for event assessment at grouped survey waves, produced corresponding HRs of 1.25, 1.19, and 1.19 (**Supplementary Table 11**). Schoenfeld-residual tests showed no evidence of proportional-hazards violations for the marker terms or globally in any of the four Cox models (all P>0.18).

The supplementary CHARLS analyses using alternative analytic frameworks are presented in **Supplementary Tables 2** and **3**. Neither the 2011 baseline analysis of 2011–2015 outcomes nor the analysis of all event-free participants with diabetes in 2015 showed significant associations. To examine this heterogeneity, the broader 2015 diabetes population was stratified by diabetes history. Among participants with diabetes already identified in 2011, TyG, TCBI, and METS-IR were positively associated with subsequent outcomes, with HRs of 1.27, 1.23, and 2.44, respectively; the same markers were not significant among participants newly identified with diabetes in 2015. Interaction P values were 0.006, 0.010, and 0.018, respectively (**Supplementary Table 12**). CHG was not significant in either subgroup.

### 3.3 Comparison with conventional glycemic markers and incremental associations beyond FBG and HbA1c

The head-to-head comparison between conventional glycemic markers and metabolic indices is shown in **Supplementary Table 4**. These analyses were based on complete-case samples of 801 participants with 240 events for FBG, HbA1c, and TyG, and 788 participants with 237 events for TCBI and METS-IR. In the same clinically adjusted model, FBG was not significantly associated with subsequent composite cardiorenal outcomes (HR, 1.10; 95% CI, 0.97-1.25; P = 0.131), whereas HbA1c was significantly associated with a higher risk of the composite outcome (HR, 1.18; 95% CI, 1.05-1.33; P = 0.005). TyG, TCBI, and METS-IR were also significantly associated with composite cardiorenal outcomes, with HRs per 1-SD increase of 1.22 (95% CI, 1.07-1.40; P = 0.003), 1.21 (95% CI, 1.05-1.38; P = 0.008), and 1.22 (95% CI, 1.07-1.40; P = 0.004), respectively.

After further adjustment for FBG and HbA1c, the associations of TyG, TCBI, and METS-IR with composite cardiorenal outcomes remained statistically significant (**Supplementary Table 5**). In these incremental complete-case analyses, the samples included 800 participants with 240 events for TyG and 787 participants with 237 events for TCBI and METS-IR. The adjusted HRs per 1-SD increase were 1.26 (95% CI, 1.06-1.49; P = 0.008) for TyG, 1.19 (95% CI, 1.03-1.37; P = 0.015) for TCBI, and 1.20 (95% CI, 1.04-1.38; P = 0.013) for METS-IR.

### 3.4 Cumulative incidence and dose-response relationships

**Figure 2:**
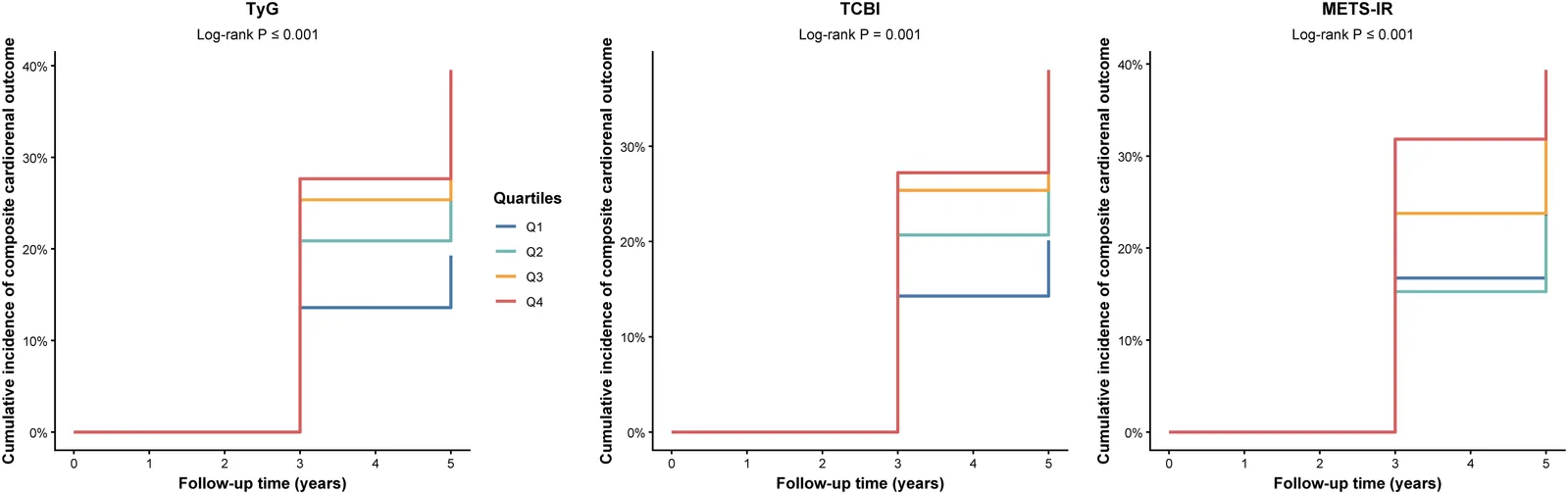
Cumulative incidence of composite cardiorenal outcomes by quartiles of metabolic indices in the principal CHARLS reassessment cohort. Kaplan-Meier curves are shown for quartiles of TyG, TCBI, and METS-IR. Event time was approximated by the survey wave of first outcome report, and differences among quartiles were assessed using log-rank tests. Q1 and Q4 denote the lowest and highest quartiles, respectively. CHARLS, China Health and Retirement Longitudinal Study; TyG, triglyceride-glucose index; TCBI, triglyceride-total cholesterol-body weight index; METS-IR, metabolic score for insulin resistance.

Kaplan-Meier curves according to quartiles of TyG, TCBI, and METS-IR are shown in **Figure 2**. The cumulative incidence of composite cardiorenal outcomes differed significantly across quartiles of all three indices, with log-rank P values of <0.001 for TyG, 0.001 for TCBI, and <0.001 for METS-IR. Higher quartiles generally showed higher cumulative incidence, particularly for TyG and METS-IR.

**Figure 3:**
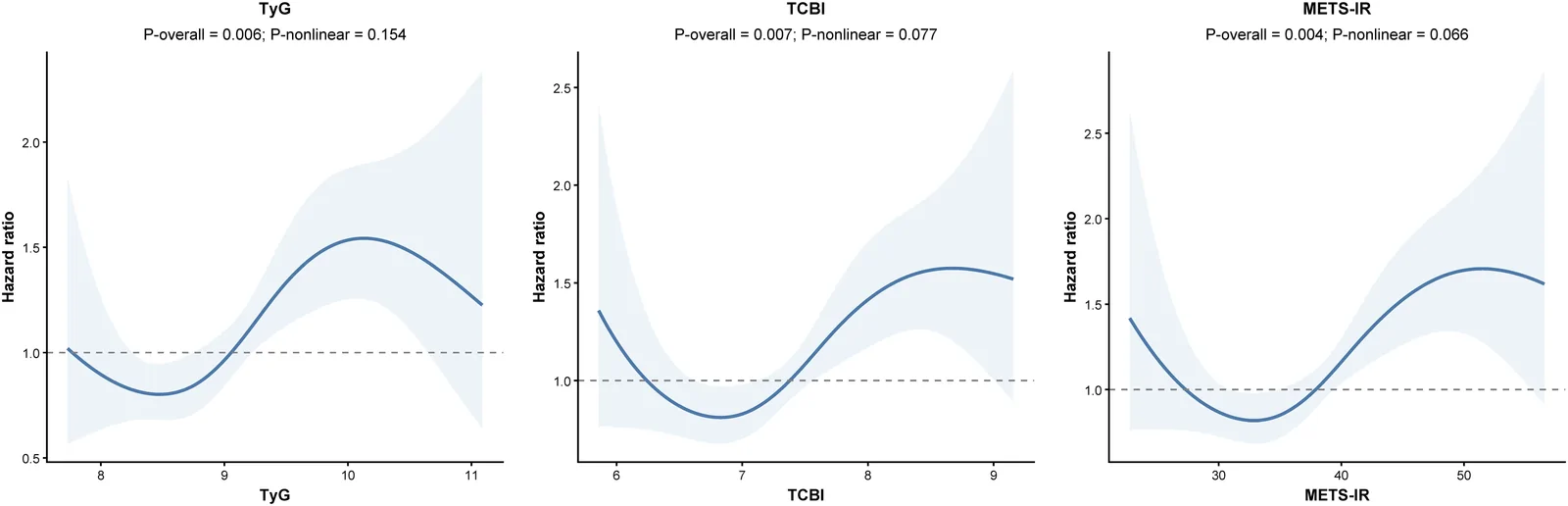
Restricted cubic spline associations of metabolic indices with composite cardiorenal outcomes in the principal CHARLS reassessment cohort. Splines with 3 degrees of freedom were fitted for TyG, log-transformed TCBI, and METS-IR over the 1st to 99th percentile of each distribution, with the median as the reference. Models were adjusted for age, sex, ever smoking, ever drinking, hypertension, marital status, residence, and education. Event time was approximated by the survey wave of first outcome report. Solid lines indicate adjusted hazard ratios, shaded areas indicate 95% confidence intervals, and the horizontal dashed line indicates a hazard ratio of 1.0. CHARLS, China Health and Retirement Longitudinal Study; TyG, triglyceride-glucose index; TCBI, triglyceride-total cholesterol-body weight index; METS-IR, metabolic score for insulin resistance.

Restricted cubic spline analyses are shown in **Figure 3**. TyG, TCBI, and METS-IR each showed significant overall associations with subsequent composite cardiorenal outcomes, with P-overall values of 0.006, 0.007, and 0.004, respectively. The corresponding P values for nonlinearity were 0.154, 0.077, and 0.066, respectively, indicating no statistically significant evidence of nonlinear associations.

The graphical analyses for CHG are presented in the **Supplementary Figures**. Cumulative incidence differed across CHG quartiles in the Kaplan-Meier analysis (**Supplementary Figure 1**; log-rank P = 0.025). However, in the restricted cubic spline analysis, CHG was not significantly associated with the composite cardiorenal outcome, and no significant nonlinearity was observed (**Supplementary Figure 2**; P-overall=0.431; P-nonlinear=0.758).

### 3.5 Discrimination and reclassification for wave-approximated 4-year composite cardiorenal outcome

**Figure 4:**
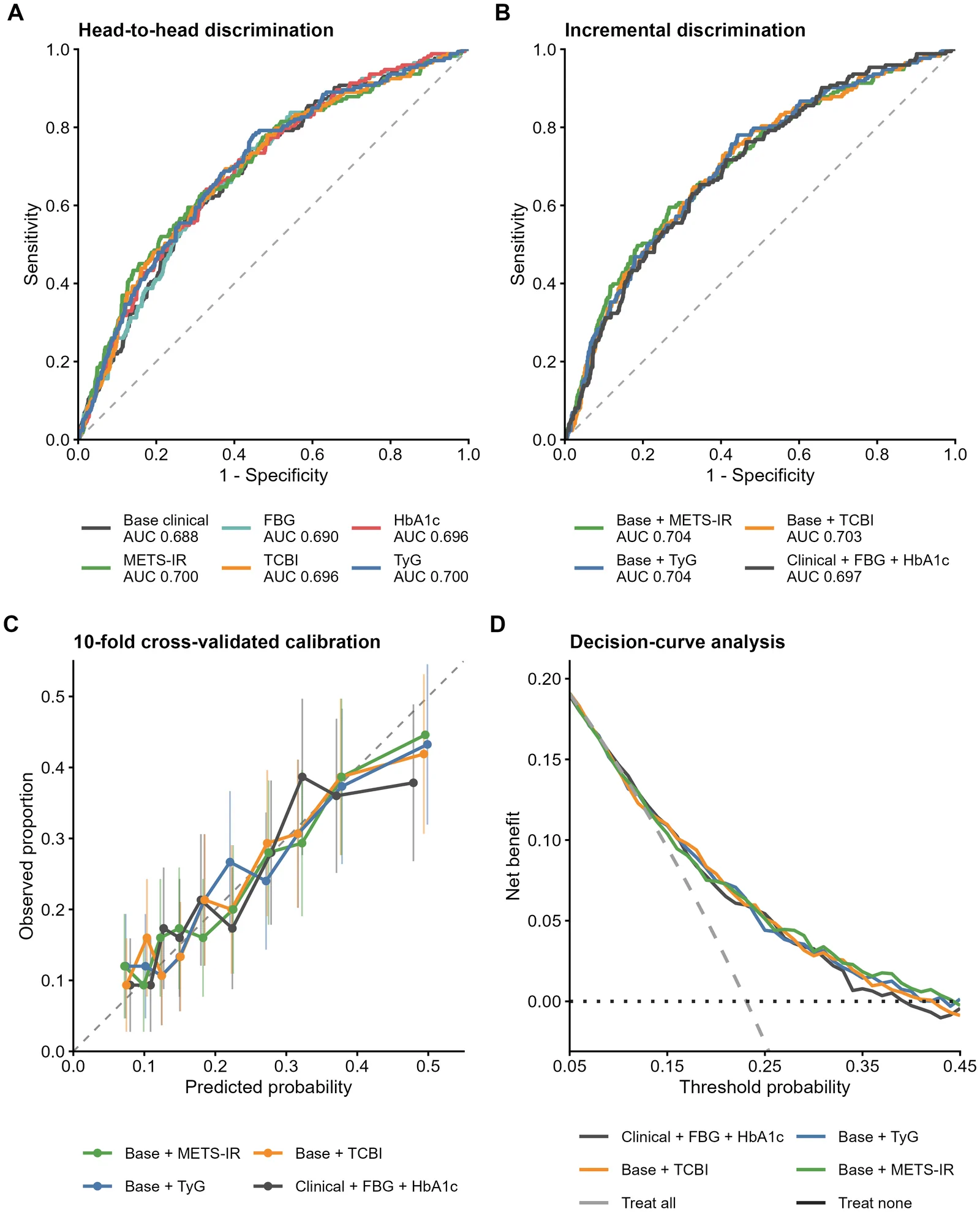
Discrimination, calibration, and decision-curve analyses for the wave-approximated 4-year composite cardiorenal outcome in CHARLS. All panels used the same complete-case sample (N = 749; 173 events). The clinical model included age, sex, ever smoking, ever drinking, hypertension, marital status, residence, and education. **(A)** compares apparent ROC curves for the clinical model and models separately adding FBG, HbA1c, TyG, TCBI, or METS-IR. **(B)** compares apparent ROC curves for the reference model containing clinical variables, FBG, and HbA1c and models additionally containing TyG, TCBI, or METS-IR. **(C)** shows decile-based calibration from 10-fold cross-validated predictions; points indicate observed proportions, vertical bars indicate approximate 95% confidence intervals, and the dashed line indicates perfect calibration. **(D)** shows decision curves based on the same out-of-fold predictions across threshold probabilities of 0.05-0.45; treat-all and treat-none strategies are shown for reference. CHARLS, China Health and Retirement Longitudinal Study; ROC, receiver operating characteristic; AUC, area under the ROC curve; FBG, fasting blood glucose; HbA1c, glycated hemoglobin; TyG, triglyceride-glucose index; TCBI, triglyceride-total cholesterol-body weight index; METS-IR, metabolic score for insulin resistance.

The discrimination and reclassification analyses used a common prediction sample of 749 participants with 173 events (**Figure 4**; **Table 3**; **Supplementary Tables 6, 13**). Apparent AUCs were 0.688 for the clinical model, 0.690 after adding FBG, 0.696 after adding HbA1c, 0.700 after adding TyG, 0.696 after adding TCBI, and 0.700 after adding METS-IR (**Figure 4A**). After TyG, TCBI, or METS-IR was added to the model containing clinical variables, FBG, and HbA1c, apparent AUCs were 0.704, 0.703, and 0.704, respectively (**Figure 4B**), and the corresponding changes were not statistically significant (**Supplementary Table 6**). In 10-fold cross-validation, the AUC of the clinical, FBG, and HbA1c model was 0.665 and increased only to 0.669-0.671 after adding an index; Brier scores changed from 0.1687 to 0.1673-0.1683, and calibration slopes were 0.777-0.790 (**Figure 4C**; **Supplementary Table 13**). Decision curves showed no consistent net-benefit advantage for any index across the evaluated thresholds (**Figure 4D**).

**Table 3:** Incremental reclassification beyond FBG and HbA1c for the wave-approximated 4-year composite cardiorenal outcome in CHARLS.

Comparison	N	Events	Continuous NRI (95% CI)	P for NRI	IDI (95% CI)	P for IDI
Reference model + TyG	749	173	0.246 (0.060, 0.408)	0.004	0.008 (0.001, 0.015)	0.036
Reference model + TCBI	749	173	0.214 (0.031, 0.382)	0.024	0.006 (0.000, 0.012)	0.052
Reference model + METS-IR	749	173	0.245 (0.068, 0.399)	<0.001	0.010 (0.003, 0.017)	0.020

The reference model included age, sex, ever smoking, ever drinking, hypertension, marital status, residence, education, FBG, and HbA1c. Extended models additionally included TyG, TCBI, or METS-IR. Continuous NRI denotes category-free net reclassification improvement; IDI denotes integrated discrimination improvement. Confidence intervals and P values were estimated using 500 bootstrap resamples. All comparisons used the same complete-case sample with a valid wave-approximated 4-year outcome classification. FBG, fasting blood glucose; HbA1c, glycated hemoglobin; TyG, triglyceride-glucose index; TCBI, triglyceride-total cholesterol-body weight index; METS-IR, metabolic score for insulin resistance; NRI, net reclassification improvement; IDI, integrated discrimination improvement; CI, confidence interval.

Reclassification analyses are presented in **Table 3**. Adding TyG yielded a continuous NRI of 0.246 (95% CI, 0.060-0.408; P = 0.004) and an IDI of 0.008 (95% CI, 0.001-0.015; P = 0.036). Adding TCBI yielded a continuous NRI of 0.214 (95% CI, 0.031-0.382; P = 0.024) and an IDI of 0.006 (95% CI, 0.000-0.012; P = 0.052). Adding METS-IR yielded a continuous NRI of 0.245 (95% CI, 0.068-0.399; P<0.001) and an IDI of 0.010 (95% CI, 0.003-0.017; P = 0.020). These apparent reclassification measures were interpreted together with the cross-validated discrimination, calibration, and decision-curve results.

### 3.6 Subgroup analyses and individual cardiovascular and kidney outcomes

**Figure 5:**
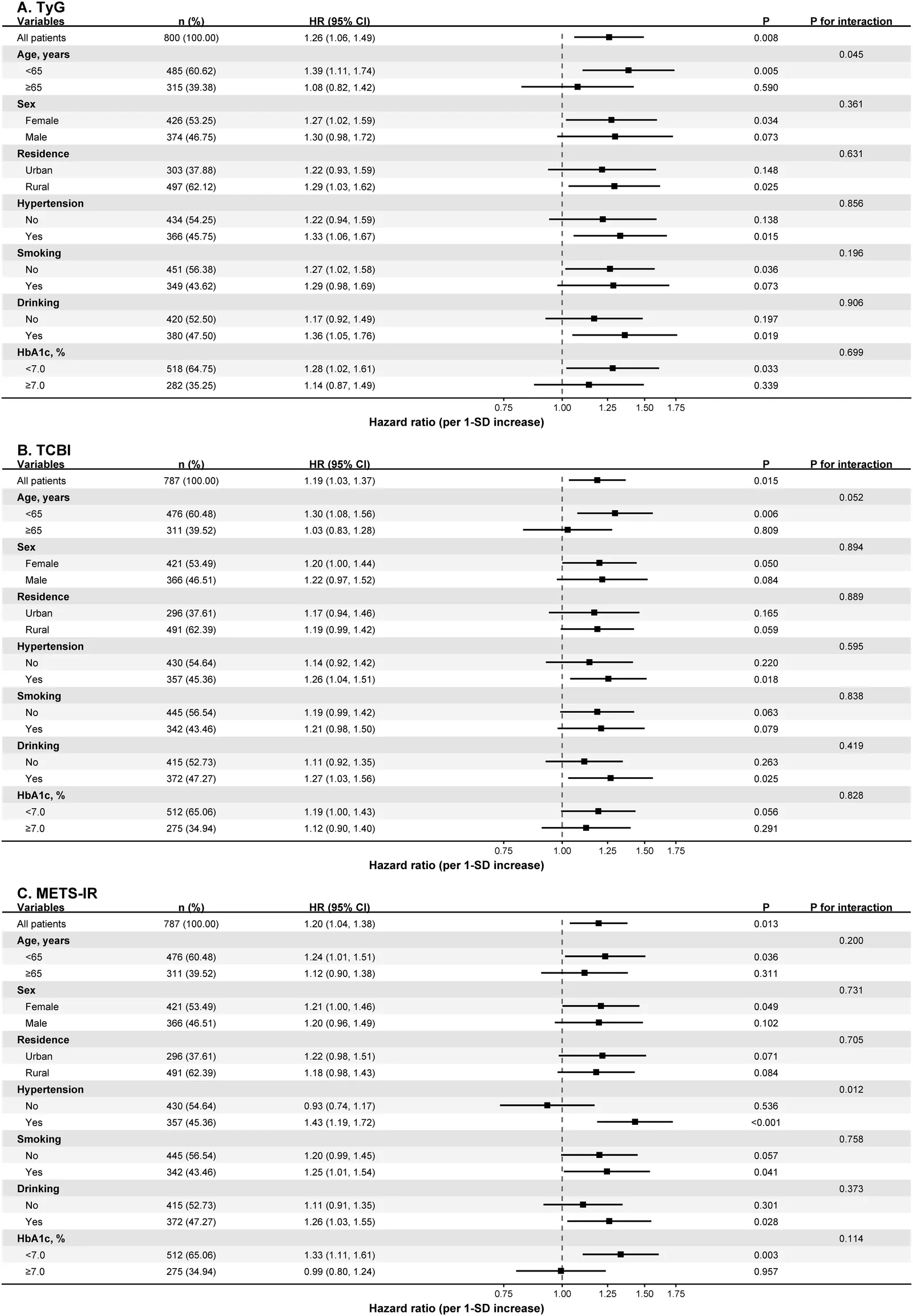
Subgroup analyses of metabolic indices and composite cardiorenal outcomes in the principal CHARLS reassessment cohort. Panels A-C show forest plots of hazard ratios per 1-SD increase in TyG (A), TCBI (B), and METS-IR (C) across prespecified subgroups. Models were adjusted for age, sex, ever smoking, ever drinking, hypertension, marital status, residence, education, FBG, and HbA1c, except that the variable defining a subgroup was omitted from adjustment for that subgroup. P values for interaction were obtained by adding marker-by-subgroup interaction terms to the Cox models. CHARLS, China Health and Retirement Longitudinal Study; SD, standard deviation; FBG, fasting blood glucose; HbA1c, glycated hemoglobin; TyG, triglyceride-glucose index; TCBI, triglyceride-total cholesterol-body weight index; METS-IR, metabolic score for insulin resistance; HR, hazard ratio; CI, confidence interval.

Subgroup analyses for TyG, TCBI, and METS-IR are shown in **Figure 5**. For TyG, the association with composite cardiorenal outcomes differed by age group (P for interaction=0.045), with a significant association among participants aged <65 years but not among those aged ≥65 years. For TCBI, no statistically significant interaction was observed across the prespecified subgroups. For METS-IR, a significant interaction was observed by hypertension status (P for interaction=0.012), with a significant association among participants with hypertension but not among those without hypertension. Across the remaining subgroup variables, no statistically significant interactions were observed.

Associations with individual cardiovascular and kidney outcomes are presented in **Table 4**. TyG was significantly associated with both cardiovascular outcomes (HR, 1.17; 95% CI, 1.01–1.36; P = 0.036) and kidney outcomes (HR, 1.35; 95% CI, 1.07–1.70; P = 0.012). TCBI and METS-IR were significantly associated with cardiovascular outcomes, with HRs of 1.17 (95% CI, 1.00–1.36; P = 0.045) and 1.23 (95% CI, 1.06–1.42; P = 0.007), respectively. Neither TCBI nor METS-IR was significantly associated with kidney outcomes.

**Table 4:** Associations of metabolic indices with individual cardiovascular and kidney outcomes in the principal CHARLS reassessment cohort.

Marker	Cardiovascular outcome				Kidney outcome			
	N	Events	HR (95% CI)	P	N	Events	HR (95% CI)	P
TyG	801	198	1.17 (1.01, 1.36)	0.036	801	73	1.35 (1.07, 1.70)	0.012
TCBI	788	196	1.17 (1.00, 1.36)	0.045	788	72	1.24 (0.97, 1.58)	0.091
METS-IR	788	196	1.23 (1.06, 1.42)	0.007	788	72	1.20 (0.93, 1.53)	0.155

Models were adjusted for age, sex, ever smoking, ever drinking, hypertension, marital status, residence, and education; estimates are presented per 1-SD increase. TCBI was log-transformed before standardization. Outcome-specific complete-case samples were used, so sample sizes and event counts differ from those of the principal eligible cohort. SD, standard deviation; HR, hazard ratio; CI, confidence interval; TCBI, triglyceride-total cholesterol-body weight index. TyG, triglyceride-glucose index; METS-IR, metabolic score for insulin resistance.

In additional exploratory analyses, VAI and LAP were positively associated with the composite outcome in CHARLS, with HRs per 1-SD increase of 1.13 and 1.19, respectively (**Supplementary Table 14**). Replacing ever smoking with current smoking did not materially alter the results for TyG, TCBI, or METS-IR. No sex interaction was detected for any index in CHARLS; in the hospital cohort, the TCBI-sex interaction was significant (P = 0.026), whereas the interactions for TyG, CHG, and METS-IR were not (**Supplementary Table 15**).

### 3.7 Independent hospital validation cohort

The associations between metabolic indices and composite cardiorenal outcomes during post-landmark follow-up in the hospital cohort are shown in **Table 5**. In the multiply imputed glycemia-adjusted Model 4, TyG, CHG, TCBI, and METS-IR were associated with higher composite-outcome risk, with HRs per 1-SD increase of 1.25 (95% CI, 1.09-1.42; P = 0.001), 1.22 (95% CI, 1.01-1.47; P = 0.040), 1.26 (95% CI, 1.11-1.42; P<0.001), and 1.18 (95% CI, 1.04-1.34; P = 0.008), respectively. The CHG estimate was weaker and less consistent with the graphical analyses than the estimates for the other indices.

**Table 5:** Associations of metabolic indices with subsequent composite cardiorenal outcomes during post-landmark follow-up in the hospital validation cohort.

Categories	Model 1		Model 2		Model 3		Model 4	
	HR (95% CI)	P	HR (95% CI)	P	HR (95% CI)	P	HR (95% CI)	P
TyG								
Continuous	1.27 (1.13, 1.43)	<0.001	1.25 (1.11, 1.40)	<0.001	1.23 (1.10, 1.39)	<0.001	1.25 (1.09, 1.42)	<0.001
Q1	Ref		Ref		Ref		Ref	
Q2	1.48 (1.03, 2.12)	0.033	1.48 (1.04, 2.12)	0.031	1.45 (1.01, 2.09)	0.042	1.48 (1.03, 2.13)	0.035
Q3	1.97 (1.40, 2.77)	<0.001	1.92 (1.37, 2.71)	<0.001	1.88 (1.33, 2.65)	<0.001	1.92 (1.34, 2.75)	<0.001
Q4	1.99 (1.40, 2.82)	<0.001	1.87 (1.32, 2.67)	<0.001	1.82 (1.27, 2.60)	<0.001	1.86 (1.27, 2.73)	0.001
CHG								
Continuous	1.13 (1.01, 1.27)	0.036	1.16 (1.03, 1.30)	0.015	1.16 (1.03, 1.30)	0.015	1.22 (1.01, 1.47)	0.040
Q1	Ref		Ref		Ref		Ref	
Q2	0.98 (0.70, 1.38)	0.906	1.00 (0.71, 1.41)	0.989	1.00 (0.71, 1.40)	0.992	1.05 (0.73, 1.50)	0.808
Q3	1.21 (0.87, 1.69)	0.251	1.26 (0.91, 1.76)	0.168	1.26 (0.90, 1.75)	0.177	1.35 (0.91, 2.01)	0.133
Q4	1.40 (1.02, 1.94)	0.038	1.47 (1.06, 2.03)	0.021	1.46 (1.05, 2.02)	0.023	1.61 (1.01, 2.55)	0.044
TCBI								
Continuous	1.31 (1.17, 1.48)	<0.001	1.26 (1.12, 1.43)	<0.001	1.24 (1.09, 1.41)	<0.001	1.26 (1.11, 1.42)	<0.001
Q1	Ref		Ref		Ref		Ref	
Q2	1.70 (1.19, 2.42)	0.003	1.64 (1.15, 2.34)	0.007	1.61 (1.12, 2.31)	0.009	1.67 (1.17, 2.39)	0.005
Q3	1.86 (1.31, 2.66)	<0.001	1.74 (1.22, 2.49)	0.002	1.69 (1.17, 2.45)	0.005	1.75 (1.23, 2.50)	0.002
Q4	2.42 (1.69, 3.46)	<0.001	2.16 (1.50, 3.12)	<0.001	2.09 (1.43, 3.07)	<0.001	2.15 (1.49, 3.10)	<0.001
METS-IR								
Continuous	1.24 (1.10, 1.40)	<0.001	1.19 (1.05, 1.35)	0.005	1.37 (1.05, 1.79)	0.020	1.18 (1.04, 1.34)	0.008
Q1	Ref		Ref		Ref		Ref	
Q2	1.80 (1.27, 2.56)	<0.001	1.75 (1.23, 2.49)	0.002	1.88 (1.29, 2.76)	0.001	1.74 (1.22, 2.47)	0.002
Q3	1.72 (1.21, 2.45)	0.002	1.60 (1.12, 2.29)	0.009	1.83 (1.17, 2.87)	0.008	1.57 (1.10, 2.24)	0.014
Q4	2.14 (1.50, 3.06)	<0.001	1.92 (1.33, 2.77)	<0.001	2.41 (1.33, 4.37)	0.004	1.87 (1.29, 2.71)	<0.001

Model 1 adjusted for age and sex. Model 2 additionally adjusted for smoking, drinking, and hypertension. Model 3 included Model 2 covariates plus BMI. Model 4 included Model 2 covariates plus FBG and HbA1c and is referred to as the glycemia-adjusted model. Models 3 and 4 are presented separately because BMI or body weight is a component of, or closely related to, several metabolic indices. Continuous estimates are shown per 1-SD increase; quartile analyses use Q1 as the reference. TCBI was log-transformed before standardization and modeling. Missing smoking, drinking, and hypertension covariates were imputed in 20 datasets, and estimates were pooled using Rubin’s rules. Follow-up began at the 90-day landmark and was censored at the first event, the last available hospital record, or 4 years after the landmark, whichever occurred first. BMI, body mass index; FBG, fasting blood glucose; HbA1c, glycated hemoglobin; SD, standard deviation; Q, quartile; HR, hazard ratio; CI, confidence interval; TCBI, triglyceride-total cholesterol-body weight index. TyG, triglyceride-glucose index; CHG, cholesterol-high-density lipoprotein-glucose index; METS-IR, metabolic score for insulin resistance.

In quartile-based analyses, Q4 of each index was associated with higher risk than Q1 in glycemia-adjusted Model 4. The HRs were 1.86 (95% CI, 1.27-2.73; P = 0.001) for TyG, 1.61 (95% CI, 1.01-2.55; P = 0.044) for CHG, 2.15 (95% CI, 1.49-3.10; P<0.001) for TCBI, and 1.87 (95% CI, 1.29-2.71; P = 0.001) for METS-IR (**Table 5**).

**Figure 6:**
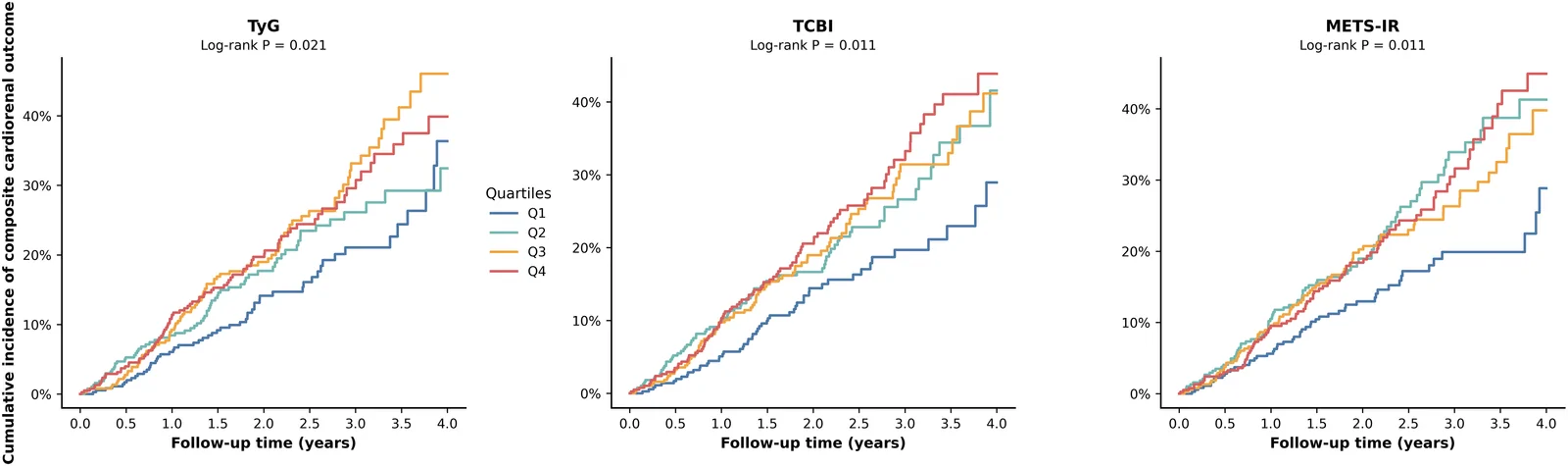
Cumulative incidence of composite cardiorenal outcomes by quartiles of metabolic indices in the hospital validation cohort. Kaplan-Meier curves are shown for quartiles of TyG, TCBI, and METS-IR during follow-up after the 90-day landmark. Follow-up was censored at the first event, the last available hospital record, or 4 years after the landmark, whichever occurred first. Differences among quartiles were assessed using log-rank tests. Q1 and Q4 denote the lowest and highest quartiles, respectively. TyG, triglyceride-glucose index; TCBI, triglyceride-total cholesterol-body weight index; METS-IR, metabolic score for insulin resistance.

Kaplan-Meier analyses in the hospital validation cohort showed significant differences in cumulative incidence across quartiles of TyG, TCBI, and METS-IR (**Figure 6**; log-rank P = 0.021, 0.011, and 0.011, respectively). In the complete supplementary KM analyses including all four indices, CHG did not show a significant difference across quartiles (**Supplementary Figure 3**). Restricted cubic spline analyses showed significant overall associations for TyG, TCBI, and METS-IR, without statistically significant nonlinearity; CHG was not significantly associated with the composite outcome in the spline analysis (**Supplementary Figure 4**).

For individual component outcomes in the hospital cohort, TyG, CHG, TCBI, and METS-IR were associated with cardiovascular outcomes. TyG and TCBI were also associated with kidney outcomes, whereas CHG and METS-IR were not (**Supplementary Table 8**). In IPCW time-dependent analyses, the AUC of the clinical model with FBG and HbA1c was 0.625, 0.653, 0.628, and 0.640 at 1, 2, 3, and approximately 4 years, respectively. Adding an index changed AUC by 0.003-0.024, with no statistically significant comparison (**Supplementary Figure 5**). Horizon-specific AUCs were non-monotonic; because each horizon used different case-control sets and censoring weights, they were interpreted as discrete estimates rather than a continuous performance trajectory. The approximately 4-year estimate had limited support because only 67 event-free patients remained observed beyond that horizon.

## 4 Discussion

In this study of adults with diabetes identified in 2011 who attended the 2015 CHARLS reassessment and remained free of overt cardiorenal outcomes, reassessment-stage TyG, TCBI, and METS-IR were associated with subsequent composite cardiorenal outcomes after adjustment including FBG and HbA1c. The estimates were similar after selection weighting and in discrete-time models, and proportional-hazards diagnostics did not identify violations. TyG showed the most consistent pattern across continuous, quartile, and component-outcome analyses. An independent single-center hospital validation cohort provided supportive clinical replication for TyG, TCBI, and METS-IR, whereas CHG showed a weaker and less coherent pattern across regression and graphical analyses. Prediction gains beyond clinical variables, FBG, and HbA1c remained small. Together, these findings support a potential role for simple metabolic indices as low-burden adjunctive markers of residual cardiorenal vulnerability at reassessment, rather than as stand-alone clinical prediction tools.

A central feature of the present study is the focus on reassessment-stage risk stratification rather than conventional prediction from the time of diabetes identification alone. Patients with diabetes are not static risk carriers; their cardiorenal risk evolves over time with aging, glycemic exposure, adiposity, blood pressure, lipid remodeling, kidney function, treatment, and lifestyle change. Contemporary cardiorenal and diabetes guidelines emphasize repeated assessment and dynamic management of cardiovascular and kidney risk rather than reliance on a single historical measurement (7–10, 40). From this perspective, our design addresses a clinically distinct question: among individuals with diabetes who have survived and remained free of overt cardiorenal outcomes by a later reassessment point, do their current metabolic indices provide information about subsequent risk? This reassessment framework is conceptually aligned with dynamic prediction and landmark-based thinking, in which risk is updated among individuals who are still at risk at a clinically meaningful time point (29, 41). Therefore, the present findings should not be interpreted simply as baseline prediction from diabetes onset, but as evidence that reassessment-stage metabolic status may help refine risk stratification during ongoing diabetes care.

The null findings in the two alternative CHARLS analyses and the observed selection differences define the boundary of the inference. Participants entering the principal cohort were younger and less likely to have hypertension at the 2011 baseline than those not included. IPSW substantially improved balance in measured baseline variables, and the weighted estimates remained similar to the primary results, suggesting that measured selection factors alone did not explain the associations. However, weighting cannot remove the conditioning inherent in surviving, returning for reassessment, and remaining event-free through 2015, nor can it eliminate bias from unmeasured selection factors. The diabetes-history analysis further showed that positive associations were concentrated among participants with diabetes already identified in 2011 and were absent among those newly identified in 2015. This pattern is compatible with a reassessment-stage signal in longer-standing diabetes, but it also indicates heterogeneity and possible survivor- or collider-type selection. The results should therefore not be generalized to all adults with diabetes or to prediction from initial diabetes identification.

The findings are also consistent with the emerging cardiovascular-kidney-metabolic framework, which views diabetes, obesity, kidney dysfunction, and cardiovascular disease as interconnected rather than isolated domains (5, 6, 40). Insulin resistance and related metabolic disturbances may contribute through triglyceride-rich lipoprotein metabolism, endothelial dysfunction, oxidative stress, chronic low-grade inflammation, adipose tissue dysfunction, and pro-inflammatory and pro-fibrotic signaling (11, 12, 42). Exploratory CHARLS analyses of VAI and LAP also showed positive associations with subsequent outcomes, supporting a role for adiposity-related metabolic burden. Because waist circumference was unavailable in the hospital analytic file, these exploratory findings could not be replicated there. The evaluated indices remain clinical surrogates rather than direct mechanistic measures, and the observational results do not establish causality.

Although TyG, TCBI, METS-IR, and CHG combine routinely available measurements, their performance suggests overlapping but non-identical domains. TyG, derived from triglycerides and fasting glucose, showed the most coherent CHARLS pattern (13–15, 21–26). METS-IR incorporates BMI and HDL-C and may reflect a broader adiposity- and dyslipidemia-related phenotype (16, 27, 28), whereas TCBI may capture a lipid-body-mass or nutritional-metabolic dimension (17, 18). CHG was null in the glycemia-adjusted CHARLS model but weakly significant in the multiply imputed hospital model, while its hospital Kaplan-Meier and spline analyses were not significant. Accordingly, CHG was considered less consistent rather than uniformly negative. Differences across indices may reflect population characteristics, endpoint definitions, metabolic-index construction, and timing of measurement.

The independent hospital cohort adds an important but complementary layer of evidence. The direction and statistical consistency of TyG, TCBI, and METS-IR were reproduced despite differences in setting, patient profile, outcome ascertainment, and follow-up. Because each marker was standardized using its own within-cohort SD, cross-cohort HR magnitudes are not directly equivalent. Exact hospital dates also allowed censoring-aware time-dependent AUC analysis, which showed only small, non-significant discrimination gains. The non-monotonic horizon-specific AUC pattern should not be interpreted as a temporal gain or loss in model performance because case-control composition and censoring weights changed across horizons; later estimates were also less precise as follow-up support diminished. Thus, the hospital cohort supports replication of association direction but does not constitute definitive external validation of a clinical prediction model. Its single-center retrospective design, substantial analytic exclusions, and limited support near 4 years remain important constraints.

The prediction analyses should be interpreted with appropriate caution. Apparent AUC increments were small and statistically non-significant, and 10-fold cross-validation showed similarly small changes in AUC and Brier score. Calibration slopes of approximately 0.78 suggested some overfitting, while decision curves did not show a consistent net-benefit advantage across thresholds. Continuous NRI and small IDI values indicated movement in predicted risks but, because continuous NRI is sensitive to clinically trivial changes, these metrics cannot establish clinical utility on their own (33). The indices should therefore not replace established risk assessment. Their practical value lies in being low-burden adjunctive markers that may help flag residual metabolic vulnerability for closer reassessment, a role that still requires testing against clinically actionable thresholds and prospective management decisions.

Subgroup and component-outcome analyses provided additional context. The prespecified CHARLS subgroup results were generally directionally consistent, although the age interaction for TyG and hypertension interaction for METS-IR require caution because of multiple testing. Exploratory sex-specific analyses showed no significant sex interactions for any index in CHARLS. In the hospital cohort, only the TCBI-sex interaction was significant, an exploratory finding that requires independent confirmation. Replacing ever smoking with current smoking in CHARLS did not materially change the primary associations. In component analyses, TyG was associated with both cardiovascular and kidney outcomes in CHARLS; in the hospital cohort, TyG and TCBI were associated with both components, whereas METS-IR and CHG were associated with cardiovascular but not kidney outcomes.

This study has several strengths. It used a nationally representative CHARLS cohort with standardized biomarker assessment, a clinically motivated reassessment-stage design, multiple complementary analyses, and supportive replication in an independent hospital cohort with exact record dates. Selection weighting, discrete-time modeling, proportional-hazards diagnostics, cross-validated calibration, decision curves, and IPCW time-dependent AUC analyses directly tested important assumptions. Several limitations remain. First, CHARLS outcomes were survey reported and exact onset dates were unavailable. Event timing was therefore grouped by survey wave; although discrete-time complementary log-log results agreed with Cox estimates, interval uncertainty remains. Kidney outcomes lacked repeated eGFR or albuminuria trajectories and neither cohort provided renal histopathology, so fibrosis or glomerulosclerosis was not assessed. Second, restriction to survivors who returned for reassessment and remained event-free may induce selection or collider-type bias. IPSW addressed measured selection factors but not the target-population conditioning itself or unmeasured determinants. Third, CHARLS used model-specific complete-case analyses, whereas the hospital cohort used multiple imputation for three non-core covariates; this difference and cohort-specific standardization limit direct numerical comparison. Fourth, diabetes etiology, smoking dose, and detailed medication use were unavailable, including complete information on SGLT2 inhibitors and GLP-1 receptor agonists. Fifth, the fixed-horizon CHARLS prediction sample excluded participants without sufficient outcome information, and the hospital cohort had limited follow-up support near 4 years. Finally, the hospital cohort was single-center and retrospective, and residual confounding remains possible. The findings should therefore be interpreted as reassessment-stage associations rather than causal effects or a validated clinical prediction tool.

## 5 Conclusion

Among adults with diabetes identified in 2011 who attended the 2015 CHARLS reassessment and remained free of overt cardiorenal outcomes, higher reassessment-stage TyG, TCBI, and METS-IR were associated with subsequent composite cardiorenal outcomes. Similar estimates after selection weighting and discrete-time modeling, together with supportive direction in an independent hospital validation cohort, strengthen the evidence that these indices capture residual metabolic vulnerability in this reassessment population. However, their incremental predictive performance was limited, and the findings cannot be generalized to all diabetes populations. These routinely available indices may be considered exploratory adjunctive markers for reassessment-stage risk evaluation, pending further multicenter validation of calibration, thresholds, and clinical impact.

## Data Availability

Publicly available datasets were analyzed in this study. The CHARLS dataset is publicly available from the China Health and Retirement Longitudinal Study data platform: https://charls.pku.edu.cn/. Access to CHARLS requires registration and approval by the CHARLS research team. No accession number is applicable. The independent hospital validation cohort is not publicly available because it contains de-identified clinical patient data subject to institutional and patient privacy restrictions. Requests for access to de-identified hospital validation data may be directed to the corresponding authors and are subject to approval by the relevant institution and ethics committee.
