# Supplementary material for: Reassessment-stage metabolic indices and subsequent cardiorenal outcomes in adults with diabetes: CHARLS and an independent hospital validation cohort
Source: Front Endocrinol (Lausanne). 2026 Aug 28;17:1926018. doi: 10.3389/fendo.2026.1926018 (PMC13561846; doi:10.3389/fendo.2026.1926018)
Supplement: Supplementary file 1 [file Supplementaryfile1.docx]

**Supplementary Figure S1. Cumulative incidence of composite cardiorenal outcomes by CHG quartiles in the principal CHARLS reassessment cohort.** Kaplan-Meier curves are shown according to CHG quartiles. Event time was approximated by the survey wave of first outcome report, and differences among quartiles were assessed using the log-rank test. Q1 and Q4 denote the lowest and highest quartiles, respectively. Abbreviations: CHARLS, China Health and Retirement Longitudinal Study; CHG, cholesterol-high-density lipoprotein-glucose index.

**Supplementary Figure S2. Restricted cubic spline association of CHG with composite cardiorenal outcomes in the principal CHARLS reassessment cohort.** A spline with 3 degrees of freedom was fitted over the 1st to 99th percentile of the CHG distribution, with the median as the reference. The model was adjusted for age, sex, ever smoking, ever drinking, hypertension, marital status, residence, and education. Event time was approximated by the survey wave of first outcome report. The solid line indicates the adjusted hazard ratio, the shaded area indicates the 95% confidence interval, and the horizontal dashed line indicates a hazard ratio of 1.0. Abbreviations: CHARLS, China Health and Retirement Longitudinal Study; CHG, cholesterol-high-density lipoprotein-glucose index.

**Supplementary Figure S3. Cumulative incidence of composite cardiorenal outcomes by quartiles of four metabolic indices in the hospital validation cohort.** Kaplan-Meier curves are shown for quartiles of TyG, CHG, TCBI, and METS-IR during follow-up after the 90-day landmark. Follow-up was censored at the first event, the last available hospital record, or 4 years after the landmark, whichever occurred first. Differences among quartiles were assessed using log-rank tests. Q1 and Q4 denote the lowest and highest quartiles, respectively. Abbreviations: TyG, triglyceride-glucose index; CHG, cholesterol-high-density lipoprotein-glucose index; TCBI, triglyceride-total cholesterol-body weight index; METS-IR, metabolic score for insulin resistance.

**Supplementary Figure S4. Restricted cubic spline associations of metabolic indices with composite cardiorenal outcomes in the hospital validation cohort.** Splines with 3 degrees of freedom were fitted for TyG, CHG, log-transformed TCBI, and METS-IR over the 1st to 99th percentile of each distribution, with the median as the reference. Complete-case models were adjusted for age, sex, smoking, drinking, and hypertension. Follow-up began at the 90-day landmark and was censored at the first event, the last available hospital record, or 4 years after the landmark, whichever occurred first. Solid lines indicate adjusted hazard ratios, shaded areas indicate 95% confidence intervals, and horizontal dashed lines indicate a hazard ratio of 1.0. Abbreviations: TyG, triglyceride-glucose index; CHG, cholesterol-high-density lipoprotein-glucose index; TCBI, triglyceride-total cholesterol-body weight index; METS-IR, metabolic score for insulin resistance.

**Supplementary Figure S5. IPCW time-dependent AUCs for composite cardiorenal outcomes in the hospital validation cohort.** Points and error bars show horizon-specific AUC estimates and pointwise 95% confidence intervals at 1, 2, 3, and approximately 4 years after the 90-day landmark. Values in parentheses beneath the time points indicate the number of event-free patients observed beyond each horizon. The reference model included age, sex, smoking, drinking, hypertension, FBG, and HbA1c; extended models additionally included TyG, TCBI, or METS-IR. Linear predictors were averaged across 20 multiply imputed datasets. Abbreviations: IPCW, inverse probability of censoring weighting; AUC, area under the receiver operating characteristic curve; FBG, fasting blood glucose; HbA1c, glycated hemoglobin; TyG, triglyceride-glucose index; TCBI, triglyceride-total cholesterol-body weight index; METS-IR, metabolic score for insulin resistance.

**Supplementary Table S1. Composition of the CHARLS analysis datasets**

| **Supplementary analysis dataset** | **Baseline survey wave** | **Participants with diabetes at baseline-definition stage** | **Event-free at baseline** | **Re-assessed at 2015 (if applicable)** | **Final analytic sample** | **Composite cardiorenal events** |
| --- | --- | --- | --- | --- | --- | --- |
| Participants with diabetes already present in 2011, re-assessed in 2015, free of cardiorenal events at 2015, and followed after 2015 | 2015 | 2343 | 1206 | 1913 | 1104 | 311 |
| Participants with diabetes at the 2011 baseline, free of cardiorenal events at 2011, and followed for incident cardiorenal outcomes through 2015 | 2011 | 2343 | 1694 |  | 1575 | 222 |
| All participants with diabetes at the 2015 survey, free of cardiorenal events at 2015, and followed after 2015 | 2015 | 3302 | 1878 |  | 1728 | 527 |

At the relevant survey wave, diabetes was defined by self-reported diabetes, FBG ≥126 mg/dL, or HbA1c ≥6.5%. The datasets were constructed for distinct analysis questions and are not mutually exclusive. Abbreviations: CHARLS, China Health and Retirement Longitudinal Study; FBG, fasting blood glucose; HbA1c, glycated hemoglobin.

**Supplementary Table S2. Associations of metabolic indices with composite cardiorenal outcomes from 2011 to 2015 among participants with diabetes at the 2011 baseline**

| **Marker** | **N** | **Events** | **HR (95% CI)** | **P value** |
| --- | --- | --- | --- | --- |
| TyG | 1385 | 190 | 0.92 (0.79, 1.07) | 0.269 |
| CHG | 1385 | 190 | 0.96 (0.83, 1.11) | 0.551 |
| TCBI | 1187 | 161 | 0.93 (0.79, 1.10) | 0.398 |
| METS-IR | 1181 | 159 | 0.95 (0.79, 1.15) | 0.615 |

Models were adjusted for age, sex, ever smoking, ever drinking, hypertension, marital status, residence, and education. Hazard ratios are presented per 1-SD increase in each index. TCBI was log-transformed before standardization and modeling. Analyses used model-specific complete-case samples. Abbreviations: SD, standard deviation; HR, hazard ratio; CI, confidence interval; TCBI, triglyceride-total cholesterol-body weight index. Additional abbreviations: TyG, triglyceride-glucose index; CHG, cholesterol-high-density lipoprotein-glucose index; METS-IR, metabolic score for insulin resistance.

**Supplementary Table S3. Associations of metabolic indices with post-2015 composite cardiorenal outcomes among all event-free participants with diabetes in 2015**

| **Marker** | **N** | **Events** | **HR (95% CI)** | **P value** |
| --- | --- | --- | --- | --- |
| TyG | 1469 | 450 | 1.01 (0.92, 1.12) | 0.778 |
| CHG | 1469 | 450 | 0.98 (0.89, 1.08) | 0.661 |
| TCBI | 1438 | 441 | 1.05 (0.95, 1.16) | 0.337 |
| METS-IR | 1435 | 439 | 0.93 (0.74, 1.15) | 0.488 |

Models were adjusted for age, sex, ever smoking, ever drinking, hypertension, marital status, residence, and education. Hazard ratios are presented per 1-SD increase in each index. TCBI was log-transformed before standardization and modeling. Analyses used model-specific complete-case samples. Abbreviations: SD, standard deviation; HR, hazard ratio; CI, confidence interval; TCBI, triglyceride-total cholesterol-body weight index. Additional abbreviations: TyG, triglyceride-glucose index; CHG, cholesterol-high-density lipoprotein-glucose index; METS-IR, metabolic score for insulin resistance.

**Supplementary Table S4. Head-to-head comparison of conventional glycemic markers and metabolic indices in the principal CHARLS reassessment cohort**

| **Marker** | **N** | **Events** | **HR (95% CI)** | **P value** |
| --- | --- | --- | --- | --- |
| FBG | 801 | 240 | 1.10 (0.97, 1.25) | 0.131 |
| HbA1c | 801 | 240 | 1.18 (1.05, 1.33) | 0.005 |
| TyG | 801 | 240 | 1.22 (1.07, 1.40) | 0.003 |
| TCBI | 788 | 237 | 1.21 (1.05, 1.38) | 0.008 |
| METS-IR | 788 | 237 | 1.22 (1.07, 1.40) | 0.004 |

Each marker was evaluated in a separate model adjusted for age, sex, ever smoking, ever drinking, hypertension, marital status, residence, and education. Hazard ratios are presented per 1-SD increase. TCBI was log-transformed before standardization and modeling. Analyses used marker-specific complete-case samples. Abbreviations: FBG, fasting blood glucose; HbA1c, glycated hemoglobin; SD, standard deviation; HR, hazard ratio; CI, confidence interval; TCBI, triglyceride-total cholesterol-body weight index. Additional abbreviations: TyG, triglyceride-glucose index; CHG, cholesterol-high-density lipoprotein-glucose index; METS-IR, metabolic score for insulin resistance.

**Supplementary Table S5. Associations of metabolic indices beyond FBG and HbA1c in the principal CHARLS reassessment cohort**

| **Marker** | **N** | **Events** | **HR (95% CI)** | **P value** |
| --- | --- | --- | --- | --- |
| TyG | 800 | 240 | 1.26 (1.06, 1.49) | 0.008 |
| TCBI | 787 | 237 | 1.19 (1.03, 1.37) | 0.015 |
| METS-IR | 787 | 237 | 1.20 (1.04, 1.38) | 0.013 |

Models were adjusted for age, sex, ever smoking, ever drinking, hypertension, marital status, residence, education, FBG, and HbA1c. Hazard ratios are presented per 1-SD increase in each index. TCBI was log-transformed before standardization and modeling. Analyses used marker-specific complete-case samples. Abbreviations: FBG, fasting blood glucose; HbA1c, glycated hemoglobin; SD, standard deviation; HR, hazard ratio; CI, confidence interval; TCBI, triglyceride-total cholesterol-body weight index. Additional abbreviations: TyG, triglyceride-glucose index; METS-IR, metabolic score for insulin resistance.

**Supplementary Table S6. Changes in AUC after adding metabolic indices beyond FBG and HbA1c for the wave-approximated 4-year composite cardiorenal outcome in CHARLS**

| **Comparison** | **N** | **Events** | **Reference AUC** | **New AUC** | **ΔAUC** | **P for ΔAUC** |
| --- | --- | --- | --- | --- | --- | --- |
| Reference model + TyG | 749 | 173 | 0.697 | 0.704 | 0.007 | 0.344 |
| Reference model + TCBI | 749 | 173 | 0.697 | 0.703 | 0.005 | 0.407 |
| Reference model + METS-IR | 749 | 173 | 0.697 | 0.704 | 0.007 | 0.340 |

The reference model included age, sex, ever smoking, ever drinking, hypertension, marital status, residence, education, FBG, and HbA1c. Extended models additionally included TyG, TCBI, or METS-IR. Paired DeLong tests were used to compare AUCs. All comparisons used the same complete-case sample with a valid wave-approximated 4-year outcome classification. Abbreviations: AUC, area under the receiver operating characteristic curve; CI, confidence interval; FBG, fasting blood glucose; HbA1c, glycated hemoglobin; TyG, triglyceride-glucose index; TCBI, triglyceride-total cholesterol-body weight index; METS-IR, metabolic score for insulin resistance.

**Supplementary Table S7. Baseline characteristics of the hospital validation cohort according to subsequent composite cardiorenal outcome**

| **Characteristic** | **Overall (N=1,578)** | **No event (N=1,282)** | **Composite event (N=296)** | **P value** |
| --- | --- | --- | --- | --- |
| Age, years | 53.0 (41.0-61.0) | 52.0 (40.0-60.0) | 55.0 (45.0-64.0) | <0.001 |
| Male sex, n (%) | 1018 (64.5) | 822 (64.1) | 196 (66.2) | 0.544 |
| Body mass index, kg/m² | 26.12 (24.10-28.90) | 25.95 (23.94-28.82) | 26.65 (24.77-29.11) | 0.009 |
| Hypertension, n (%) | 587 (42.2) | 443 (39.0) | 144 (56.5) | <0.001 |
| Current/ever smoking, n (%) | 641 (41.8) | 522 (41.7) | 119 (42.0) | 0.947 |
| Current/ever drinking, n (%) | 607 (40.7) | 478 (39.5) | 129 (45.7) | 0.059 |
| Fasting blood glucose, mmol/L | 8.70 (7.20-10.10) | 8.70 (7.20-10.10) | 8.70 (7.28-10.30) | 0.552 |
| HbA1c, % | 8.85 (7.50-10.40) | 8.90 (7.50-10.40) | 8.70 (7.60-10.30) | 0.558 |
| Triglycerides, mmol/L | 1.63 (1.14-2.40) | 1.58 (1.11-2.32) | 1.80 (1.26-2.51) | 0.002 |
| Total cholesterol, mmol/L | 5.06 (4.42-5.83) | 5.04 (4.44-5.78) | 5.13 (4.39-5.96) | 0.479 |
| HDL cholesterol, mmol/L | 1.09 (0.94-1.26) | 1.09 (0.94-1.26) | 1.09 (0.94-1.24) | 0.659 |
| eGFR, mL/min/1.73 m² | 106.8 (96.2-116.6) | 107.5 (97.2-117.7) | 103.8 (92.8-112.7) | <0.001 |
| UACR, mg/g | 6.4 (4.4-13.1) | 6.1 (4.3-11.7) | 8.4 (5.1-24.1) | <0.001 |
| TyG index | 9.32 (8.94-9.75) | 9.28 (8.91-9.73) | 9.42 (9.05-9.86) | 0.002 |
| CHG index | 5.89 (5.65-6.11) | 5.89 (5.65-6.10) | 5.94 (5.66-6.14) | 0.138 |
| TCBI | 2153 (1365-3548) | 2085 (1306-3508) | 2508 (1559-3787) | 0.002 |
| METS-IR | 43.49 (38.57-48.90) | 43.32 (38.23-48.71) | 44.61 (40.40-49.50) | 0.005 |

Values are presented as median (IQR) or n (%). P values compare participants with and without a subsequent composite cardiorenal outcome. Percentages were calculated using participants with available data for each variable as the denominator. Abbreviations: IQR, interquartile range; BMI, body mass index; FBG, fasting blood glucose; HbA1c, glycated hemoglobin; HDL-C, high-density lipoprotein cholesterol; eGFR, estimated glomerular filtration rate; UACR, urinary albumin-to-creatinine ratio; TyG, triglyceride-glucose index; CHG, cholesterol-high-density lipoprotein-glucose index; TCBI, triglyceride-total cholesterol-body weight index; METS-IR, metabolic score for insulin resistance.

**Supplementary Table S8. Associations of metabolic indices with individual cardiovascular and kidney outcomes in the hospital validation cohort**

| **Marker** | **Cardiovascular outcome** | | | | **Kidney outcome** | | | |
| --- | --- | --- | --- | --- | --- | --- | --- | --- |
|  | **N** | **Events** | **HR (95% CI)** | **P** | **N** | **Events** | **HR (95% CI)** | **P** |
| TyG | 1578 | 253 | 1.21 (1.06, 1.37) | 0.003 | 1578 | 74 | 1.34 (1.06, 1.69) | 0.015 |
| CHG | 1578 | 253 | 1.17 (1.03, 1.33) | 0.018 | 1578 | 74 | 1.11 (0.88, 1.41) | 0.370 |
| TCBI | 1578 | 253 | 1.21 (1.06, 1.38) | 0.005 | 1578 | 74 | 1.43 (1.12, 1.83) | 0.004 |
| METS-IR | 1578 | 253 | 1.21 (1.06, 1.38) | 0.005 | 1578 | 74 | 1.15 (0.90, 1.47) | 0.277 |

Models were adjusted for age, sex, smoking, drinking, and hypertension. Missing smoking, drinking, and hypertension covariates were imputed in 20 datasets, and estimates were pooled using Rubin's rules. Hazard ratios are presented per 1-SD increase in each index. TCBI was log-transformed before standardization and modeling. Abbreviations: SD, standard deviation; HR, hazard ratio; CI, confidence interval; TCBI, triglyceride-total cholesterol-body weight index. Additional abbreviations: TyG, triglyceride-glucose index; CHG, cholesterol-high-density lipoprotein-glucose index; METS-IR, metabolic score for insulin resistance.

**Supplementary Table S9. Baseline comparison of participants included in and not included in the principal CHARLS reassessment cohort**

| **Characteristic** | **Included n** | **Included value** | **Not included n** | **Not included value** | **P value** | **SMD** |
| --- | --- | --- | --- | --- | --- | --- |
| Age, years | 1104 | 58.56 (8.98) | 1217 | 62.52 (9.89) | <0.001 | -0.419 |
| Male sex | 1104 | 519 (47.0%) | 1239 | 589 (47.5%) | 0.799 | -0.011 |
| BMI, kg/m² | 942 | 25.09 (15.83) | 935 | 24.84 (4.42) | 0.640 | 0.022 |
| Hypertension | 1102 | 353 (32.0%) | 1221 | 647 (53.0%) | <0.001 | -0.424 |
| Ever smoking | 1104 | 415 (37.6%) | 1228 | 487 (39.7%) | 0.306 | -0.042 |
| Ever drinking | 1103 | 444 (40.3%) | 1226 | 441 (36.0%) | 0.033 | 0.088 |
| FBG, mg/dL | 1011 | 159.63 (59.63) | 988 | 161.22 (72.24) | 0.592 | -0.024 |
| HbA1c, % | 1007 | 6.02 (1.48) | 978 | 6.20 (1.58) | 0.008 | -0.118 |
| Triglycerides, mg/dL | 1011 | 204.18 (206.64) | 989 | 189.96 (183.94) | 0.104 | 0.073 |
| Total cholesterol, mg/dL | 1011 | 200.51 (45.24) | 989 | 199.76 (44.43) | 0.708 | 0.017 |
| HDL-C, mg/dL | 1011 | 45.87 (15.86) | 990 | 45.98 (15.74) | 0.873 | -0.007 |
| TyG | 1011 | 9.36 (0.82) | 988 | 9.31 (0.85) | 0.162 | 0.063 |
| CHG | 1011 | 5.84 (0.55) | 988 | 5.82 (0.58) | 0.475 | 0.032 |
| log-TCBI | 878 | 7.50 (0.87) | 787 | 7.45 (0.85) | 0.235 | 0.058 |
| METS-IR | 874 | 41.74 (25.87) | 779 | 41.24 (11.07) | 0.603 | 0.025 |

Included participants were those who entered the final principal CHARLS reassessment cohort; not-included participants were the remaining participants who met the diabetes definition in 2011. Continuous variables are presented as mean (SD), and binary variables as n (%). Denominators vary according to data availability. SMDs are calculated as included minus not included. Abbreviations: CHARLS, China Health and Retirement Longitudinal Study; SD, standard deviation; SMD, standardized mean difference. Additional abbreviations: TyG, triglyceride-glucose index; CHG, cholesterol-high-density lipoprotein-glucose index; TCBI, triglyceride-total cholesterol-body weight index; METS-IR, metabolic score for insulin resistance.

**Supplementary Table S10. Inverse-probability-of-selection weighting diagnostics and glycemia-adjusted Cox analyses in CHARLS**

Panel A. Covariate balance before and after weighting

| **Variable** | **Full 2011 diabetes frame** | **Selected cohort** | **Weighted selected cohort** | **SMD before** | **SMD after** |
| --- | --- | --- | --- | --- | --- |
| Age, years | 60.632 | 58.558 | 60.300 | -0.215 | -0.034 |
| Male sex | 0.473 | 0.470 | 0.466 | -0.006 | -0.015 |
| BMI, kg/m² | 24.863 | 24.998 | 24.871 | 0.013 | 0.001 |
| Hypertension | 0.427 | 0.320 | 0.419 | -0.216 | -0.016 |
| Ever smoking | 0.385 | 0.376 | 0.376 | -0.019 | -0.018 |
| Ever drinking | 0.378 | 0.402 | 0.378 | 0.050 | -0.000 |
| Marital status code | 0.874 | 0.904 | 0.880 | 0.090 | 0.019 |
| Rural residence | 0.548 | 0.627 | 0.557 | 0.158 | 0.019 |
| Education level code | 1.982 | 1.942 | 1.974 | -0.037 | -0.007 |
| FBG, mg/dL | 157.475 | 158.008 | 158.515 | 0.009 | 0.017 |
| HbA1c, % | 6.029 | 5.981 | 6.040 | -0.034 | 0.008 |
| Triglycerides, mg/dL | 188.562 | 198.643 | 188.682 | 0.055 | 0.001 |
| Total cholesterol, mg/dL | 199.535 | 200.132 | 199.302 | 0.014 | -0.006 |
| HDL-C, mg/dL | 45.657 | 45.720 | 45.704 | 0.004 | 0.003 |

Panel B. Glycemia-adjusted unweighted and selection-weighted Cox estimates

| **Marker** | **Unweighted HR (95% CI)** | **P value** | **IPSW HR (95% CI)** | **P value** |
| --- | --- | --- | --- | --- |
| TyG | 1.26 (1.06, 1.49) | 0.008 | 1.26 (1.05, 1.51) | 0.013 |
| CHG | 1.00 (0.79, 1.27) | 0.981 | 1.00 (0.78, 1.29) | 0.993 |
| TCBI | 1.19 (1.03, 1.37) | 0.015 | 1.18 (1.02, 1.38) | 0.027 |
| METS-IR | 1.20 (1.04, 1.38) | 0.013 | 1.23 (1.06, 1.42) | 0.006 |

Panel C. Selection-weight distribution and effective sample size

| **Median (range)** | **1st-99th percentile** | **Effective sample size** |
| --- | --- | --- |
| 0.847 (0.576-3.144) | 0.576-3.140 | 931.1 |

Binary-variable means are proportions; marital status and education are represented by survey codes. Stabilized weights were trimmed at the 1st and 99th percentiles. Cox models adjusted for age, sex, ever smoking, ever drinking, hypertension, marital status, residence, education, FBG, and HbA1c; weighted models used robust standard errors. Estimates are per 1-SD increase; TCBI was log-transformed before standardization. Abbreviations: CHARLS, China Health and Retirement Longitudinal Study; BMI, body mass index; FBG, fasting blood glucose; HbA1c, glycated hemoglobin; HDL-C, high-density lipoprotein cholesterol; IPSW, inverse-probability-of-selection weighting; SMD, standardized mean difference; HR, hazard ratio; CI, confidence interval; SD, standard deviation. Additional abbreviations: TyG, triglyceride-glucose index; CHG, cholesterol-high-density lipoprotein-glucose index; TCBI, triglyceride-total cholesterol-body weight index; METS-IR, metabolic score for insulin resistance.

**Supplementary Table S11. Discrete-time sensitivity analyses and proportional-hazards diagnostics in the principal CHARLS reassessment cohort**

| **Marker** | **Discrete-time HR (95% CI)** | **P value** | **IPSW discrete-time HR (95% CI)** | **P value** | **Marker PH P** | **Global PH P** |
| --- | --- | --- | --- | --- | --- | --- |
| TyG | 1.25 (1.06, 1.49) | 0.010 | 1.26 (1.05, 1.51) | 0.014 | 0.833 | 0.704 |
| CHG | 1.00 (0.79, 1.27) | 0.996 | 1.00 (0.77, 1.28) | 0.983 | 0.684 | 0.673 |
| TCBI | 1.19 (1.03, 1.36) | 0.016 | 1.18 (1.02, 1.37) | 0.028 | 0.869 | 0.746 |
| METS-IR | 1.19 (1.03, 1.37) | 0.015 | 1.23 (1.06, 1.42) | 0.006 | 0.187 | 0.659 |

Complementary log-log models used person-period data, survey-interval indicators, an offset for log interval length, and participant-clustered robust standard errors. Models were adjusted for age, sex, ever smoking, ever drinking, hypertension, marital status, residence, education, FBG, and HbA1c; weighted models additionally used stabilized selection weights. PH P values were obtained from Schoenfeld-residual tests for the corresponding Cox models. Estimates are per 1-SD increase, and TCBI was log-transformed before standardization. Abbreviations: CHARLS, China Health and Retirement Longitudinal Study; FBG, fasting blood glucose; HbA1c, glycated hemoglobin; IPSW, inverse-probability-of-selection weighting; PH, proportional hazards; HR, hazard ratio; CI, confidence interval; SD, standard deviation. Additional abbreviations: TyG, triglyceride-glucose index; CHG, cholesterol-high-density lipoprotein-glucose index; TCBI, triglyceride-total cholesterol-body weight index; METS-IR, metabolic score for insulin resistance.

**Supplementary Table S12. Associations stratified by diabetes history among event-free participants with diabetes in 2015**

| **Marker** | **Newly identified N/events** | **Newly identified HR (95% CI)** | **P value** | **Prevalent in 2011 N/events** | **Prevalent in 2011 HR (95% CI)** | **P value** | **P for interaction** |
| --- | --- | --- | --- | --- | --- | --- | --- |
| TyG | 755/206 | 0.91 (0.75, 1.10) | 0.320 | 515/184 | 1.27 (1.06, 1.53) | 0.011 | 0.006 |
| CHG | 755/206 | 0.90 (0.70, 1.15) | 0.392 | 515/184 | 1.01 (0.80, 1.28) | 0.937 | 0.314 |
| TCBI | 736/200 | 0.94 (0.80, 1.09) | 0.410 | 505/182 | 1.23 (1.05, 1.44) | 0.010 | 0.010 |
| METS-IR | 733/198 | 0.82 (0.47, 1.42) | 0.472 | 505/182 | 2.44 (1.10, 5.38) | 0.027 | 0.018 |

Participants were classified as having diabetes already identified in 2011 or as being newly identified with diabetes in 2015. Models were adjusted for age, sex, ever smoking, ever drinking, hypertension, marital status, residence, education, FBG, and HbA1c. Interaction P values were obtained from marker-by-diabetes-history terms in the combined sample. Estimates are per 1-SD increase, and TCBI was log-transformed before standardization. Analyses used marker-specific complete-case samples. Abbreviations: FBG, fasting blood glucose; HbA1c, glycated hemoglobin; SD, standard deviation; HR, hazard ratio; CI, confidence interval. Additional abbreviations: TyG, triglyceride-glucose index; CHG, cholesterol-high-density lipoprotein-glucose index; TCBI, triglyceride-total cholesterol-body weight index; METS-IR, metabolic score for insulin resistance.

**Supplementary Table S13. Prediction-sample comparison and 10-fold cross-validated model performance in CHARLS**

Panel A. Comparison of participants included in and excluded from the wave-approximated 4-year prediction sample

| **Characteristic** | **Included n** | **Included value** | **Excluded n** | **Excluded value** | **P value** | **SMD** |
| --- | --- | --- | --- | --- | --- | --- |
| Age, years | 749 | 62.74 (8.34) | 355 | 62.16 (10.20) | 0.353 | 0.064 |
| Male sex | 749 | 348 (46.5%) | 355 | 171 (48.2%) | 0.596 | -0.034 |
| BMI, kg/m² | 749 | 24.35 (3.48) | 184 | 23.73 (3.67) | 0.041 | 0.174 |
| Ever smoking | 749 | 325 (43.4%) | 355 | 154 (43.4%) | 0.997 | 0.000 |
| Ever drinking | 749 | 351 (46.9%) | 355 | 162 (45.6%) | 0.702 | 0.025 |
| Hypertension | 749 | 338 (45.1%) | 328 | 127 (38.7%) | 0.051 | 0.130 |
| FBG, mg/dL | 749 | 134.77 (62.99) | 75 | 154.19 (81.32) | 0.048 | -0.299 |
| HbA1c, % | 749 | 7.06 (1.88) | 75 | 7.21 (2.21) | 0.571 | -0.078 |
| TyG | 749 | 9.11 (0.75) | 75 | 9.28 (0.86) | 0.116 | -0.215 |
| CHG | 749 | 5.48 (0.49) | 75 | 5.64 (0.59) | 0.023 | -0.327 |
| log-TCBI | 749 | 7.40 (0.74) | 61 | 7.39 (0.87) | 0.969 | 0.006 |
| METS-IR | 749 | 38.05 (7.29) | 61 | 37.89 (7.75) | 0.878 | 0.022 |

Panel B. Apparent and 10-fold cross-validated performance

| **Model** | **N/events** | **Apparent AUC** | **Cross-validated AUC** | **Cross-validated Brier** | **Calibration intercept** | **Calibration slope** |
| --- | --- | --- | --- | --- | --- | --- |
| Clinical + FBG + HbA1c | 749/173 | 0.697 | 0.665 | 0.1687 | -0.235 | 0.790 |
| Base + TyG | 749/173 | 0.704 | 0.670 | 0.1681 | -0.249 | 0.777 |
| Base + TCBI | 749/173 | 0.703 | 0.669 | 0.1683 | -0.247 | 0.780 |
| Base + METS-IR | 749/173 | 0.704 | 0.671 | 0.1673 | -0.242 | 0.782 |

Panel A presents mean (SD) or n (%) using available data; SMD direction is included minus excluded. Panel B includes 749 complete cases (173 events). The reference model included age, sex, ever smoking/drinking, hypertension, marital status, residence, education, FBG, and HbA1c; each extended model added one index. Ten-fold cross-validation was stratified by outcome. Abbreviations: SD, standard deviation; SMD, standardized mean difference; FBG, fasting blood glucose; HbA1c, glycated hemoglobin.

**Supplementary Table S14. Exploratory adiposity indices and current-smoking sensitivity analyses in the principal CHARLS reassessment cohort**

Panel A. Exploratory VAI and LAP analyses

| **Marker** | **N** | **Events** | **HR (95% CI)** | **P value** |
| --- | --- | --- | --- | --- |
| VAI | 786 | 237 | 1.13 (1.01, 1.26) | 0.041 |
| LAP | 768 | 233 | 1.19 (1.06, 1.34) | 0.003 |

Panel B. Primary metabolic-index models using current smoking rather than ever smoking

| **Marker** | **N** | **Events** | **HR (95% CI)** | **P value** |
| --- | --- | --- | --- | --- |
| TyG | 800 | 240 | 1.26 (1.07, 1.50) | 0.007 |
| CHG | 800 | 240 | 1.01 (0.80, 1.28) | 0.940 |
| TCBI | 787 | 237 | 1.19 (1.04, 1.37) | 0.013 |
| METS-IR | 787 | 237 | 1.19 (1.04, 1.37) | 0.015 |

Panel A models evaluated VAI and LAP; Panel B replaced ever smoking with current smoking in the primary glycemia-adjusted models. All models were adjusted for age, sex, smoking, drinking, hypertension, marital status, residence, education, FBG, and HbA1c. Estimates are per 1-SD increase; TCBI was log-transformed before standardization. Analyses used marker-specific complete-case samples. Abbreviations: CHARLS, China Health and Retirement Longitudinal Study; VAI, visceral adiposity index; LAP, lipid accumulation product; FBG, fasting blood glucose; HbA1c, glycated hemoglobin; SD, standard deviation; HR, hazard ratio; CI, confidence interval. Additional abbreviations: TyG, triglyceride-glucose index; CHG, cholesterol-high-density lipoprotein-glucose index; TCBI, triglyceride-total cholesterol-body weight index; METS-IR, metabolic score for insulin resistance.

**Supplementary Table S15. Sex-specific metabolic-index distributions and marker-by-sex interaction tests in CHARLS and the hospital cohort**

Panel A. Metabolic-index distributions by sex

| **Cohort** | **Marker** | **Female mean (SD)** | **Male mean (SD)** | **P value** |
| --- | --- | --- | --- | --- |
| CHARLS | TyG | 9.23 (0.73) | 9.01 (0.77) | <0.001 |
| CHARLS | CHG | 5.54 (0.48) | 5.43 (0.51) | 0.001 |
| CHARLS | TCBI | 7.46 (0.71) | 7.32 (0.78) | 0.005 |
| CHARLS | METS-IR | 38.50 (6.97) | 37.51 (7.67) | 0.058 |
| Hospital | TyG | 9.31 (0.56) | 9.39 (0.62) | 0.015 |
| Hospital | CHG | 5.85 (0.34) | 5.89 (0.31) | 0.030 |
| Hospital | TCBI | 7.58 (0.62) | 7.79 (0.71) | <0.001 |
| Hospital | METS-IR | 42.69 (7.35) | 44.88 (7.60) | <0.001 |

Panel B. Marker-by-sex interaction tests for composite cardiorenal outcomes

| **Cohort** | **Marker** | **P for interaction** |
| --- | --- | --- |
| CHARLS | TyG | 0.362 |
| CHARLS | CHG | 0.520 |
| CHARLS | TCBI | 0.894 |
| CHARLS | METS-IR | 0.732 |
| Hospital | TyG | 0.113 |
| Hospital | CHG | 0.394 |
| Hospital | TCBI | 0.026 |
| Hospital | METS-IR | 0.250 |

Panel A reports unstandardized mean (SD); sex comparisons used Welch's t test. Panel B models included age, smoking, drinking, hypertension, FBG, and HbA1c; CHARLS models additionally included marital status, residence, and education. Hospital estimates were pooled across 20 imputations. Marker effects are per cohort-specific 1-SD increase; TCBI was log-transformed. Abbreviations: FBG, fasting blood glucose; HbA1c, glycated hemoglobin; SD, standard deviation.
